# Bioorthogonal activation of prodrugs, for the potential treatment of breast cancer, using the Staudinger reaction†

**DOI:** 10.1039/d3md00137g

**Published:** 2023-07-05

**Authors:** Madonna M. A. Mitry, Samuel Y. Boateng, Francesca Greco, Helen M. I. Osborn

**Affiliations:** a Reading School of Pharmacy, University of Reading Whiteknights Reading RG6 6AD UK f.greco@reading.ac.uk h.m.i.osborn@reading.ac.uk; b Dept. of Pharmaceutical Chemistry, Faculty of Pharmacy, Ain Shams University Cairo 11566 Egypt; c School of Biological Sciences, University of Reading Whiteknights Reading RG6 6ES UK

## Abstract

Selective prodrug activation at a tumor site is crucial to maximise the efficiency of chemotherapy approaches and minimise side effects due to off-site activation. In this paper, a new prodrug activation strategy is reported based on the bioorthogonal Staudinger reaction. The feasibility of this prodrug activation strategy was initially demonstrated using 9-azido sialic acid 4 as a trigger and two novel triphenylphosphine-modified N-mustard-PRO 10 and doxorubicin-PRO 12 prodrugs in an HPLC-monitored release study. Then, the azide reporter group was introduced on cancer cells' surfaces through metabolic glycoengineering of sialic acid-rich surface glycans using azide-modified monosaccharides (9-azido sialic acid 4, tetra-*O*-acetylated-9-azido sialic acid 5 and tetra-*O*-acetyl azidomannosamine). Next, the N-mustard-PRO 10 and doxorubicin-PRO 12 prodrugs were employed *in vitro* with the bioengineered cells, and activation of the prodrugs, which allowed selective release of the cytotoxic moiety at the tumour cell, was assessed. Release of the parent drugs from the prodrugs was shown to be dependent on the level of metabolic labelling, where tetra-*O*-acetyl azidomannosamine allowed the highest level of azide reporter generation in tumor cells and led to full recovery of the parent cytotoxic drug's potency. The selectivity of azide expression on breast cancer MCF-7 cells *versus* normal fibroblast L929 cells was also probed, with the 9-azido sialic acid and tetra-*O*-acetylated-9-azido sialic acid showing ∼17-fold higher azide expression on the former. Taken together, these data demonstrate the feasibility of the Staudinger reaction for selective activation of prodrugs targeted to the MCF-7 breast cancer cells.

## Abstract 2

A new and selective breast cancer-targeting prodrug system is described based on the Staudinger ligation bioorthogonal reaction.

## Graphical abstract

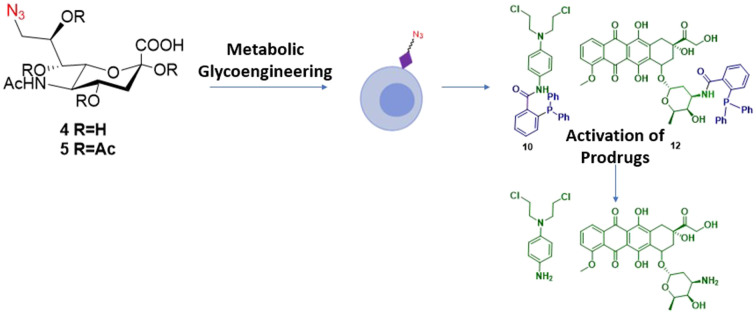

## 1. Introduction

Targeted cancer therapy is essential to address the low selectivity profiles of current chemotherapies between normal cells and cancer cells.^1,2^ For example, conventional chemotherapy drugs such as doxorubicin and nitrogen mustards are known to be highly effective chemotherapeutics for numerous cancer types including breast cancer, bladder cancer, leukaemia and lymphoma.^3–6^ However, their short-term and long-term side effects, due to their low selectivity profiles, limit their effectiveness.

Various targeted drug delivery strategies have been developed with the aim of delivering drugs selectively at tumor sites. One such strategy uses prodrugs, which rely on specific release of the active drug at the tumor site.^7,8^ The most common prodrug strategy relies on prodrug activation by local or tumor-specific enzymes either by cleavage of the cytotoxic moiety or inactivating the prodrug linker part.^9–11^ However, this enzyme-dependent activation strategy is undermined by tumor heterogeneity (non-uniformity in the level of the targeted overexpressed enzyme in all tumors), non-specific activation (*e.g.* off-site hydrolysis) and the necessity to utilize exogenous enzymes (endogenous or cytosolic enzymes require the prodrug to enter the cell).

Over the past two decades, a group of biocompatible click-chemistry reactions termed bioorthogonal reactions have been well studied and these reactions can take place *in vivo* without interfering with biological processes.^12–15^ These reactions can proceed at relatively fast reaction rates in biological conditions (aqueous environment and physiological pH) with high selectivity without interfering or interacting with other biomolecules.^16,17^ Due to their high selectivity and versatility, they have been carried out in selective targeting applications including diagnostic applications (biological imaging) and therapeutic applications (chemotherapy, immunotherapy and radioactive therapy).^15,18–20^ These applications extend to targeted prodrug activation due to the feasibility of click-and-release mechanism of some bioorthogonal reactions, however, the studies utilizing bioorthogonal reactions in prodrug activation are relatively scarce. The bioorthogonal reactions that have been utilized in bond cleavages for prodrug activation are the inverse electron demand Diels–Alder “IEDDA”^19,21^ reaction, the azide–alkene 1,3-dipolar cycloaddition reaction^22^ and Pd-mediated bond cleavage.^23^ Studies that have utilized these reactions have shown the feasibility of using the bioorthogonal cleavage reactions for prodrug activation purposes. These studies have utilized nanoparticles and antibodies for the selective delivery of bioorthogonal components to the desired site of action to then click and release the drug.

Another bioorthogonal reaction that has been utilized in selective prodrug activation is the Staudinger reaction. It is a reaction between an azide and triphenyl phosphine derivative to give an aza-ylide intermediate that, in the presence of aqueous medium, undergoes spontaneous intramolecular rearrangement to give the corresponding phosphine oxide with a stable amide linkage.^24,25^ This reaction has been reported to be utilized in prodrug activation by Azoulay *et al.*^26^ where a carbamate-linked doxorubicin/triphenylphosphine prodrug was fully activated *via* a 1,6-elimination reaction with a short-chain polymer azide trigger. The feasibility of this activation was demonstrated by HPLC release studies but no *in vitro* studies were reported. van Brakel *et al.*^27^ also reported the activation of a doxorubicin prodrug *via* the Staudinger reaction with the drug moiety being linked to the azide group rather than the triphenylphosphine. This study included *in vitro* cytotoxicity evaluation and an HPLC release study and it showed that the cytotoxicity effect and the amount of the released doxorubicin depends on the dose of the triphenylphosphine trigger added to the cells. These former studies demonstrated the potential of using the Staudinger reaction for prodrug activation applications. Herein, we aimed to realise the impact of the approach by developing a strategy that allows selective delivery of the azide trigger to cancer cells using metabolic glycoengineering (MGE).

**Fig. 1:**
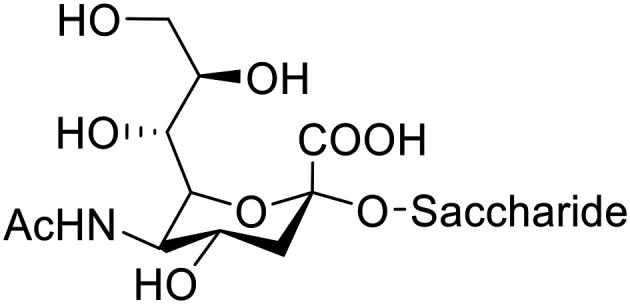
Sialic acid-component of sLe^x^, sLe^a^, sTn and PSA.

MGE involves the interception of the biosynthesis of cell surface glycans using unnatural monosaccharide precursors. These precursors carry chemical entities that are not normally found in the body to eventually express these chemical entities on the cell surface.^28–31^ In cancer, the glycosylation of the cell surfaces is changed by the overexpression of the sialyl transferase enzyme which is responsible for adding sialic acid to the terminal oligosaccharide in glycoproteins and glycolipids. This overexpression results in hypersialylation^32,33^ and the expression of tumour-associated carbohydrate antigens (TACAs). Sialic acid (*N*-acetylneuraminic acid) (Fig. 1) is reported to be one of the main components of several TACAs such as sialyl Lewis^x^ (sLe^x^), sialyl Lewis^a^ (sLe^a^), sialyl Tn (sTn) and poly sialic acid (PSA)^34^ which are overexpressed in many cancer types such as pancreatic, breast, colon, prostate and lung cancers.^32,35–37^

**Fig. 2:**
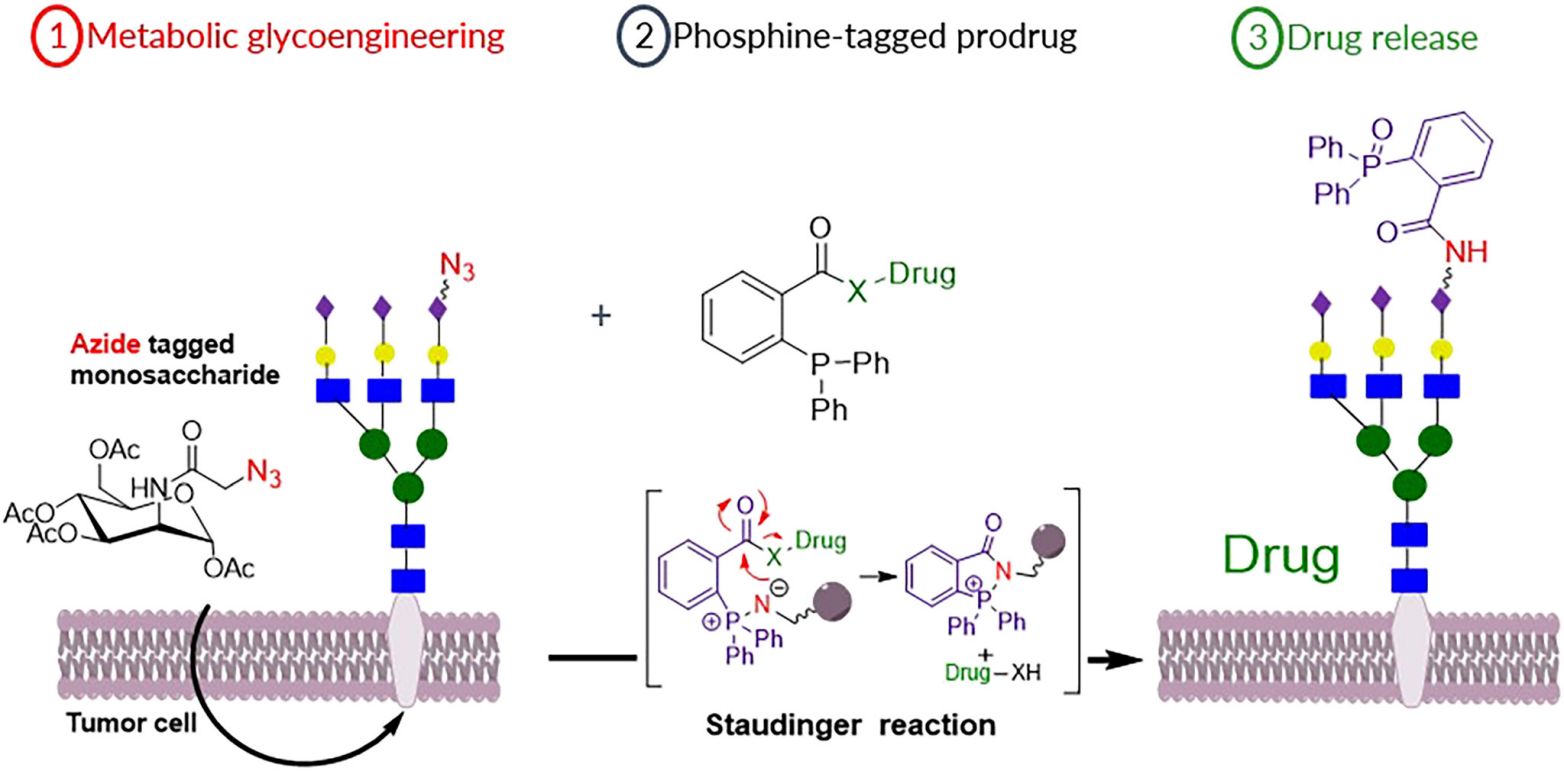
Schematic illustration of ① MGE of tumour cells by azide-modified sugar precursors to express azide functionality on its surface followed by ② targeting azide with phosphine-modified prodrugs that will cause ③ drug release by the bioorthogonal Staudinger reaction.

It has been reported that azide-modified monosaccharide precursors can interfere with the biosynthesis of polysialic acid leading to incorporation of the azidoacetyl sialic acid within cell-surface glycans.^38,39^ These surface azide reporters can then be reacted with phosphine–probe conjugates by the Staudinger bioorthogonal reaction.^40^ This approach has been used in imaging^41^ and drug delivery applications.^42^ Herein, we extend this approach to probe the feasibility of a new MGE strategy to activate prodrugs using the bioorthogonal Staudinger reaction. New azide-modified sialic acid derivatives for MGE have been synthesized for specifically labelling cancer cells with the azide functionality. The value of these for tumour specific activation of the phosphine-modified prodrugs is then demonstrated (Fig. 2).

To fully probe our approach, first, we report the design, synthesis and characterization of novel triphenylphosphine-modified nitrogen mustard 10 and doxorubicin 12 prodrugs. Also, the design, synthesis and characterization of azide-modified sialic acid derivatives 4 and 5 as monosaccharide precursors for metabolic glycoengineering is described. Then, the level of azide incorporation was evaluated by Western blotting and confocal microscopy imaging in breast cancer MCF-7 cells and mouse fibroblast L929 cells to test the selectivity of azide labelling towards them. Finally, the triphenylphosphine-modified prodrugs were tested for activation *via* Staudinger bioorthogonal reaction by the engineered azide groups *in vitro* on MCF-7 cells using the MTT assay. This is the first report detailing the biological validity of a combined MGE and bioorthogonal Staudinger reaction prodrug activation approach in MCF-7 breast cancer cells.

## 2. Results and discussion

### 2.1. Synthesis of the prodrugs and activators and proof of prodrug activation

Due to its overexpression on cell membranes of tumour cells, sialic acid was selected for functionalization with the azide moiety.^35–37^ The azide functionality can be introduced on the surface of the tumour cell through the interception of the PSA biosynthesis using azide-modified monosaccharide precursors. Azide-modified monosaccharide precursors (*i.e. N*-acetylmannosamine (ManNAc), *N*-acetylglucosamine (GlcNAc) and *N*-acetylgalactosamine (GalNAc)) were previously reported to be used for the purpose of incorporating an azide moiety among the cell surface glycans.^30,38,39,43^ However, the reports about their selectivity between cancer cells and normal cells were controversial.^44,45^

**Scheme 1:**
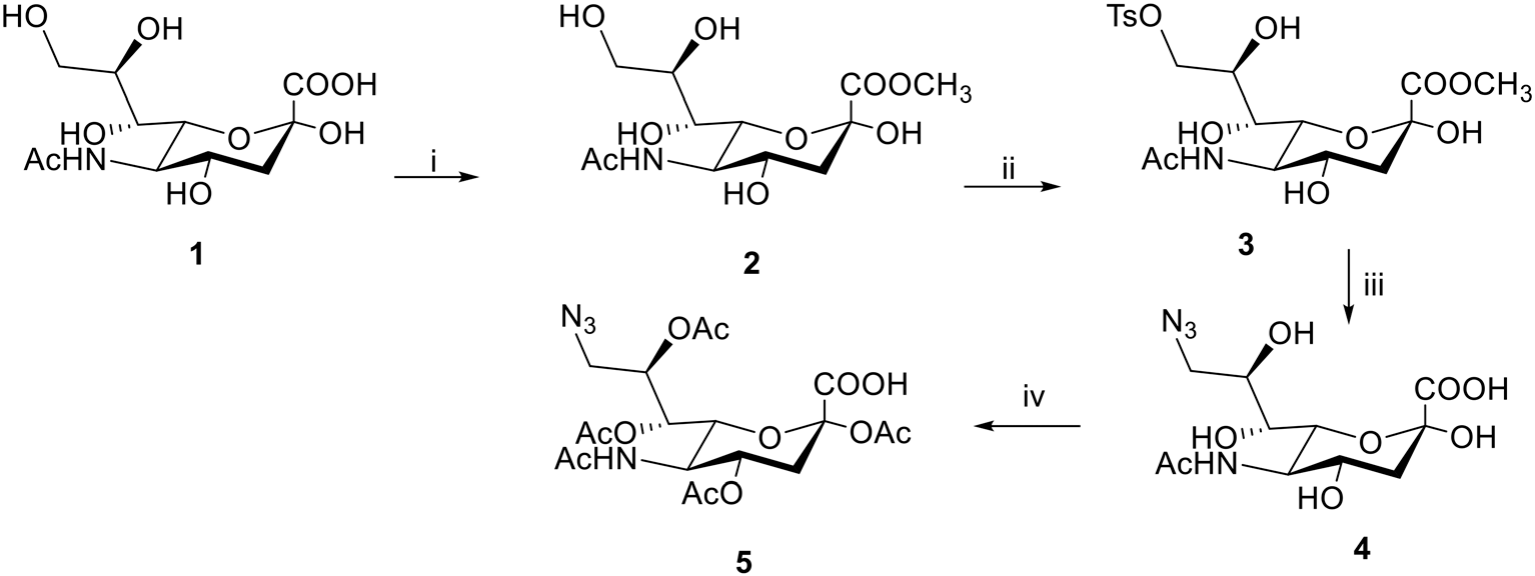
Reagents: (i) TFA, dry CH_3_OH, RT, 24 h, 86%; (ii) 4-TsCl, dry pyridine, RT, 24 h, 90%; (iii) NaN_3_ acetone, H_2_O, reflux, 24 h, 46%; (iv) Ac_2_O, DMAP, pyridine, RT, 24 h, 35%.

We developed azide-modified monosaccharides (*i.e.* sialic acid derivatives) for MGE to engineer the azide moieties on breast cancer cells' surfaces aiming to tackle the selectivity issue between the normal and cancer cells. 9-Azido sialic acids have been previously reported to successfully metabolically label surface glycans with azide functionality for applications including imaging of cell-surface sialoglycans and proteomic profiling of sialoglycoproteins. Herein, we report a new application for the azide expressed by the 9-azido sialic acid derivative namely the selective activation of triphenyl phosphine-modified prodrugs.^46^ Two 9-substituted sialic acid derivatives, specifically 9-azido-*N*-acetyl neuraminic acid 4 and 5, were synthesized according to Scheme 1.^47^

The commercially available *N*-acetyl-neuraminic acid 1 was first converted to its methyl ester 2 allowing protection of the carboxylic acid group. Then, it was converted into the tosyl derivative 3 in order to introduce a good leaving group at C-9 to be further displaced with the azide group. The first azide derivative 4 was obtained through heating the tosylated derivative 3 with sodium azide. A second azide derivative, tetra-*O*-acetylated 9-azido sialic acid 5, was synthesized since it has been proposed that acetylated derivatives can be more easily taken up by cells than the non-acetylated form.^48^ It was synthesized through acetylation of the hydroxyl groups within 9-azido sialic acid 4 with acetic anhydride.

**Scheme 2:**
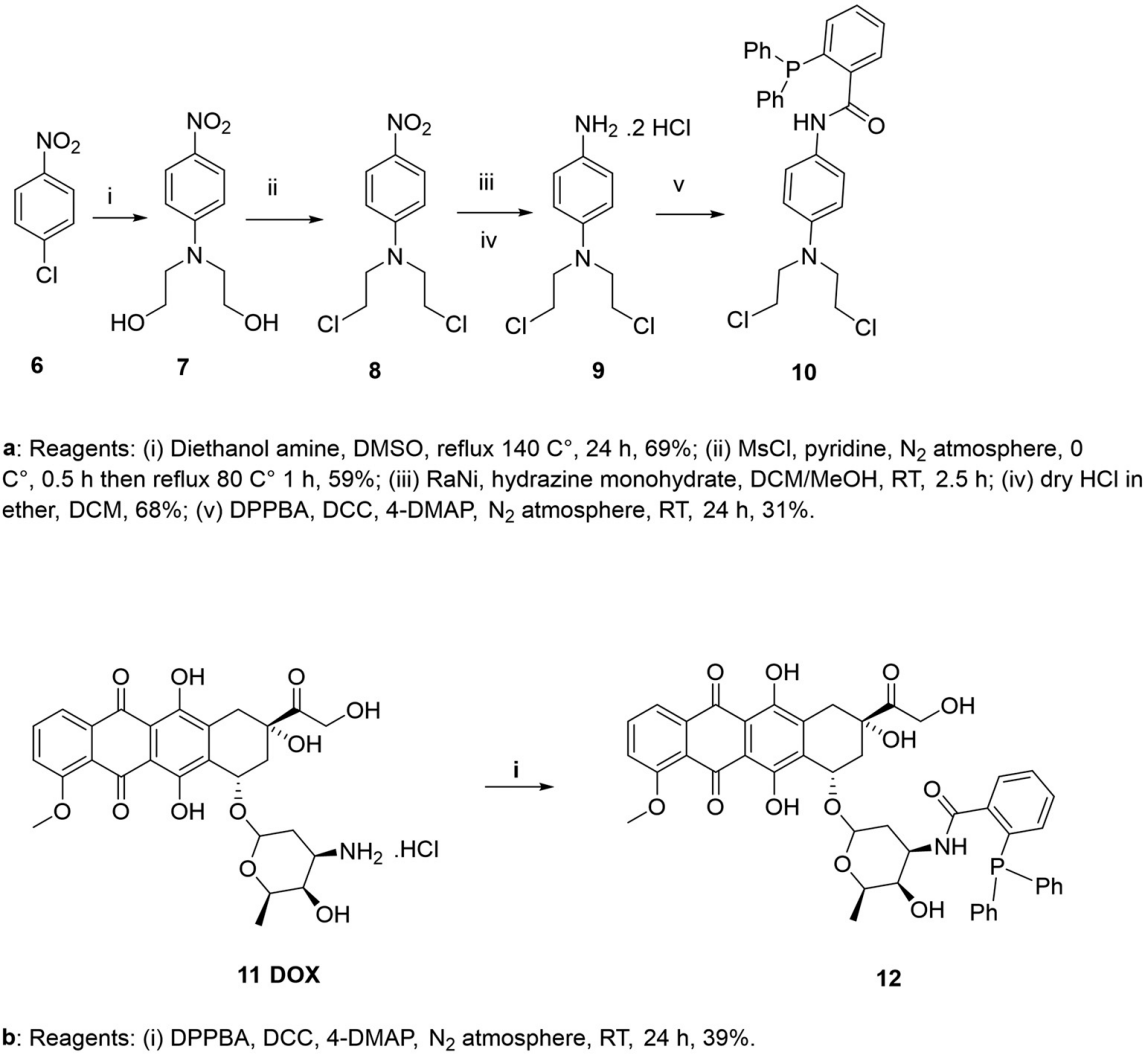
(a) Reagents: (i) diethanol amine, DMSO, reflux 140 °C, 24 h, 69%; (ii) MsCl, pyridine, N_2_ atmosphere, 0 °C, 0.5 h then reflex 80 °C, 1 h, 59%; (iii) RaNi, hydrazine monohydrate, DCM/MeOH, RT, 2.5 h; (iv) dry HCl in ether, DCM, 68%; (v) DPPBA, DCC, 4-DMAP, N_2_ atmosphere, RT 24 h, 31%. (b) Reagents: (i) DPPBA, DCC, 4-DMAP, N_2_ atmosphere, RT, 24 h, 39%.

Then, two triphenyl phosphine-modified prodrugs, *N*-(4-(bis(2-chloroethyl)amino)phenyl)-2-(diphenylphosphaneyl)benzamide 10 and 2-(diphenylphosphaneyl)-*N*-(3-hydroxy-2-methyl-6-(((1*S*,4*R*)-4,5,12-trihydroxy-4-(2-hydroxyacetyl)-10-methoxy-6,11-dioxo-1,2,3,4,6,11-hexahydrotetracen-1-yl)oxy)tetrahydro-2*H*-pyran-4-yl)benzamide 12, were synthesized according to the synthetic routes illustrated in Scheme 2a and b. Final compounds and intermediates were characterized by ^1^H NMR, ^13^C NMR and IR spectroscopy and mass spectrometry. The phosphine prodrugs were also characterized by ^31^P NMR spectroscopy and their purities confirmed by HPLC. For the synthesis of the N-mustard prodrug 10, firstly *N*,*N*-bis-(2-hydroxyethyl)-4-nitroaniline 7 was synthesized from 1-chloro-4-nitrobenzene 6 with diethanol amine. Then, the bis-alcohol derivative 7 was converted to the bis-chloro derivative 8 by mesyl chloride. Reduction of the nitro group in 8 was carried out to give the amino group which was immediately converted to the more stable HCl salt 9. Finally, the triphenyl phosphine group was introduced through dicyclohexylcarbodiimide (DCC) mediated Steglich esterification of diphenylphosphanyl benzoic acid with *N*,*N*-bis-(2-chloroethyl)benzene-1,4-diamine 9 after converting the HCl salt form to the active amino form. For the DOX prodrug, DCC-mediated amide formation of diphenylphosphanyl benzoic acid with the amino group in doxorubicin 11 resulted in the introduction of the triphenyl phosphine group.^49^

In order to test the feasibility of the Staudinger bioorthogonal reaction for prodrug activation, the release of doxorubicin 11 from doxorubicin prodrug 12, and of N-mustard from N-mustard prodrug 10, by 9-azido sialic acid 4 were monitored by HPLC for 30 hours. Full disappearance of the signals for prodrugs 10 and 12 was observed after 24 hours, however, the release profile HPLC chromatogram of the N-mustard prodrug 10 was complex due to the instability of the released N-mustard moiety (Fig. S1†). The release profile achieved by the 9-azido sialic acid 4 verified the feasibility of the prodrug activation system. This 24-hours release period can contribute to achieving sustained drug delivery through prodrug activation.^50,51^

### 2.2. *In vitro* azide-reporter generation analysis

After confirming the feasibility of the Staudinger reaction for prodrug activation, the azide-reporter generation on MCF-7 breast cancer cells and L929 fibroblast cells by two synthesized 9-azido sialic acid derivatives 4 and 5 was tested and quantified by Western blotting and confocal microscopy imaging, respectively, along with another azide-bearing metabolite precursor tetra-*O*-acetyl azidomannosamine. Tetra-*O*-acetyl azidomannosamine (Ac_4_ManNAz) has been widely reported to be used as a metabolite precursor for azide-reporter generation on cancer cells' surfaces,^38,39,45^ and it was therefore considered a relevant comparator to be used along with the two synthesized 9-azido sialic acid derivatives 4 and 5 for azide-reporter generation on MCF-7 tumor cells' surface *via* metabolic glycoengineering. First, a cell viability study for the synthesized 9-azido sialic acid derivatives 4 and 5 and Ac_4_ManNAz using a range of concentrations (1–100 μM) was conducted on MCF-7 cells to determine the highest tolerable concentration of sugar to be used subsequently in the metabolic glycoengineering in order to achieve the highest azide expression on cancer cells surfaces (Fig. S2†). At a concentration of 50 μM, the three azide-modified sugars still displayed 100% cell viability on MCF-7 cells suggesting this was an appropriate concentration to be subsequently used for metabolic glycoengineering and IC_50_ determination of the prodrugs. The same concentration (50 μM) for the three azide-modified sugars 4, 5 and Ac_4_ManNAz was also tested on mouse fibroblast cells (L929) and no change in the cells' viability was noticed compared to control (*P*-value > 0.5, 92.4 ± 1.6%, 122.4 ± 5.5% and 97.5 ± 5.2%, respectively) suggesting these sugars do not pose toxicity to normal cells (Fig. S2†).

**Fig. 3:**
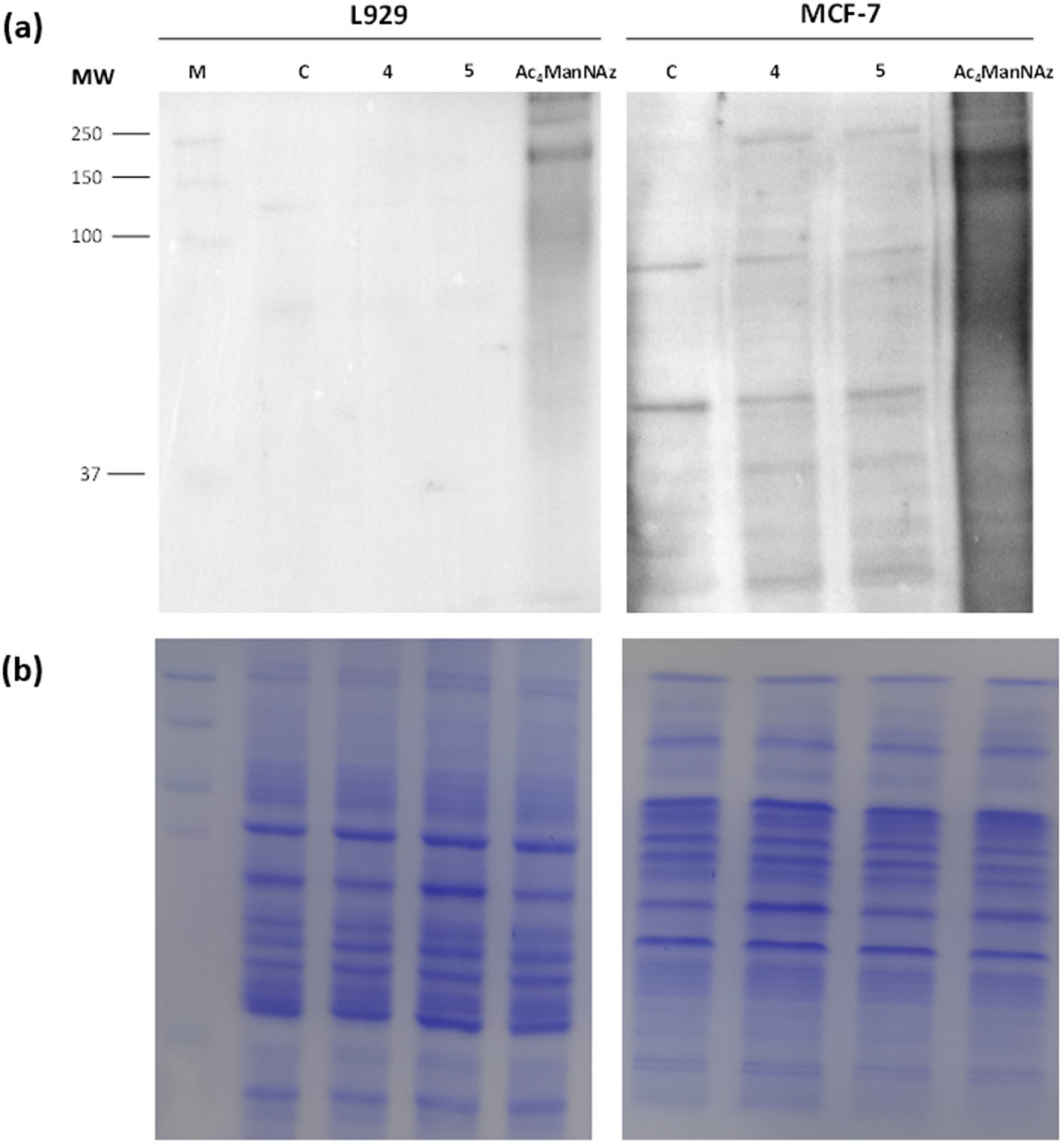
*In vitro* azide-reporter generation in glycoproteins in breast cancer cells (MCF-7) and mouse fibroblast (L929). (a) Western blot analysis of L929 fibroblasts and MCF-7 tumor cells treated separately with three azide-modified sugars Ac_4_ManNAz, 4 and 5 (50 μM). The cell lysates were reacted with phosphine-PEG_3_-biotin (500 nM) and analyzed by Western blot using an HRP-conjugated streptavidin. (b) The Coomassie staining shows the total protein loading.

Since our hypothesis relies on the difference in the metabolic rate of cancer cells compared to normal cells, and the overexpression of sialic acid within cancer cells' surfaces, the selectivity of azide expression between normal cells and cancer cells was assessed. Breast cancer cells (MCF-7) and mouse fibroblasts (L929) were incubated with 50 μM Ac_4_ManNAz, 4 and 5 for 72 h. The azide reporters' generation was analyzed by Western blotting (Fig. 3). The results showed incorporation of azide by the three sugars in the MCF-7 cells with different levels while only Ac_4_ManNAz caused the azide incorporation in L929 cells. Given that the sialic acid derivatives 4 and 5 did not show any azide-reporter generation in L929 cells, these results suggested that these derivatives 4 and 5 are more selective for azide expression on breast cancer cells rather than Ac_4_ManNAz and hence more suitable for serving the hypothesis of subsequent selective prodrug activation at tumor site.

**Fig. 4:**
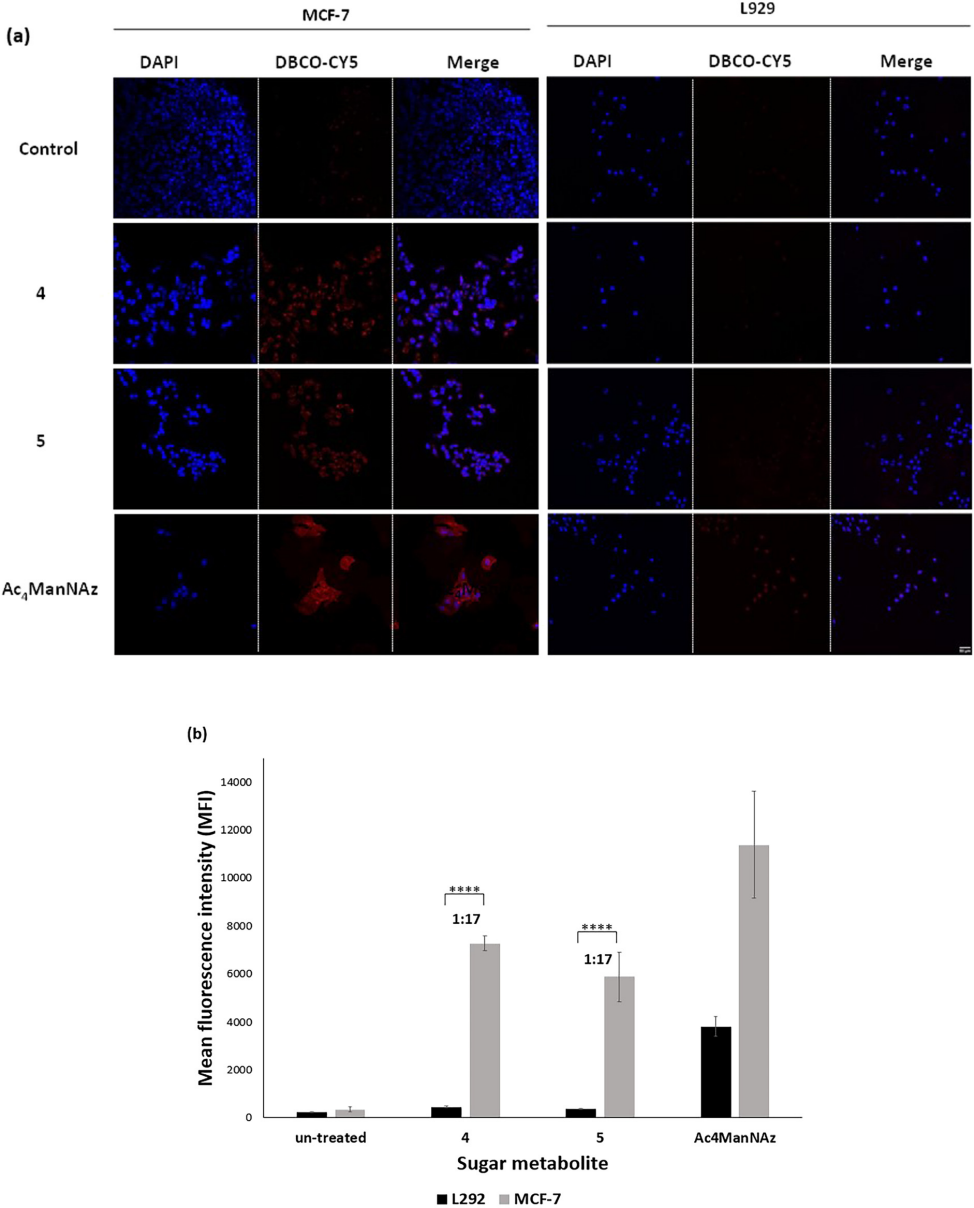
*In vitro* azide-reporter generation in breast cancer cells (MCF-7) and mouse fibroblast (L929). (a) Confocal fluorescence microscopy images of Ac_4_ManNAz, 4 and 5-treated breast cancer cells (MCF-7) and mouse fibroblast (L929), respectively (50 μM for 72 h). Azide-reporters were labelled and visualized with DBCO-Cy5 *via* bioorthogonal click reaction (20 μM) for 1 h (red-colour). Scale bar indicates 50 μm. (b) Quantification of mean fluorescence intensity (MFI) from Ac_4_ManNAz, 4 and 5-treated breast cancer cells (MCF-7) and mouse fibroblast (L929) using ImageJ software. Data are presented as mean ± SEM (*n* = 3). **** indicates difference at the *p* < 0.0001 significance level.

To further quantify the amount of azide-reporters generated on cells, relative mean fluorescence intensity (MFI) was measured by confocal microscopy imaging after incubating the Ac_4_ManNAz, 4 and 5-treated cells (MCF-7 and L929) with DBCO-Cy5 (Fig. 4 and S4†). The results confirmed the pattern previously shown by Western blotting where the three sugars showed azide incorporation and hence fluorescence in MCF-7 cells while L929 cells treated with 4 and 5 did not show any fluorescence. Quantification of the MFI showed that Ac_4_ManNAz caused azide incorporation in both L929 and MCF-7 cells with ratio 1 : 5 while 4 and 5 cause negligible azide incorporation in L929 giving a ratio of 1 : 17 compared to MCF-7 cells. These findings are important as they indicate that the activators 4 and 5 were better able to discriminate between the breast cancer cells and the normal cells than the positive control Ac_4_ManNAz.

### 2.3. *In vitro* prodrug activation and anticancer activity

After confirming the selectivity of our two 9-azido sialic acid derivatives for incorporating the azide reporter in MCF-7 cells over L929 cells, activation of the novel prodrugs 10 and 12 by the Staudinger bioorthogonal reaction was investigated in breast cancer MCF-7 cells using the MTT assay, the results are given in Table 1.

**Table 1:** IC_50_ values determined in the MCF-7 and L929 cell lines using the MTT assay. Data indicate mean ± SEM (*n* = 3)

Compound	IC_50_ (μM)	
	MCF-7	L929
Doxorubicin 11	0.2 ± 0.03	2.04 ± 0.009
Dox-prodrug 12	4.6 ± 0.19	11.3 ± 0.23
Dox-prodrug 12 on 5-engineered cells	1.6 ± 0.09	10.7 ± 0.22
Dox-prodrug 12 on 4-engineered cells	0.5 ± 0.03	11.2 ± 1.04
Dox-prodrug 12 on Ac_4_ManNAz-engineered cells	0.2 ± 0.01	7.7 ± 0.26
N-mustard prodrug 10	20.8 ± 1.27	31.4 ± 0.44
N-mustard prodrug 10 on 5-engineered cells	9.3 ± 0.63	29.7 ± 1.20
N-mustard prodrug 10 on 4-engineered cells	2.1 ± 1.11	29.9 ± 0.69
N-mustard prodrug 10 on Ac_4_ManNAz-engineered cells	0.6 ± 0.08	21.1 ± 0.58

**Fig. 5:**
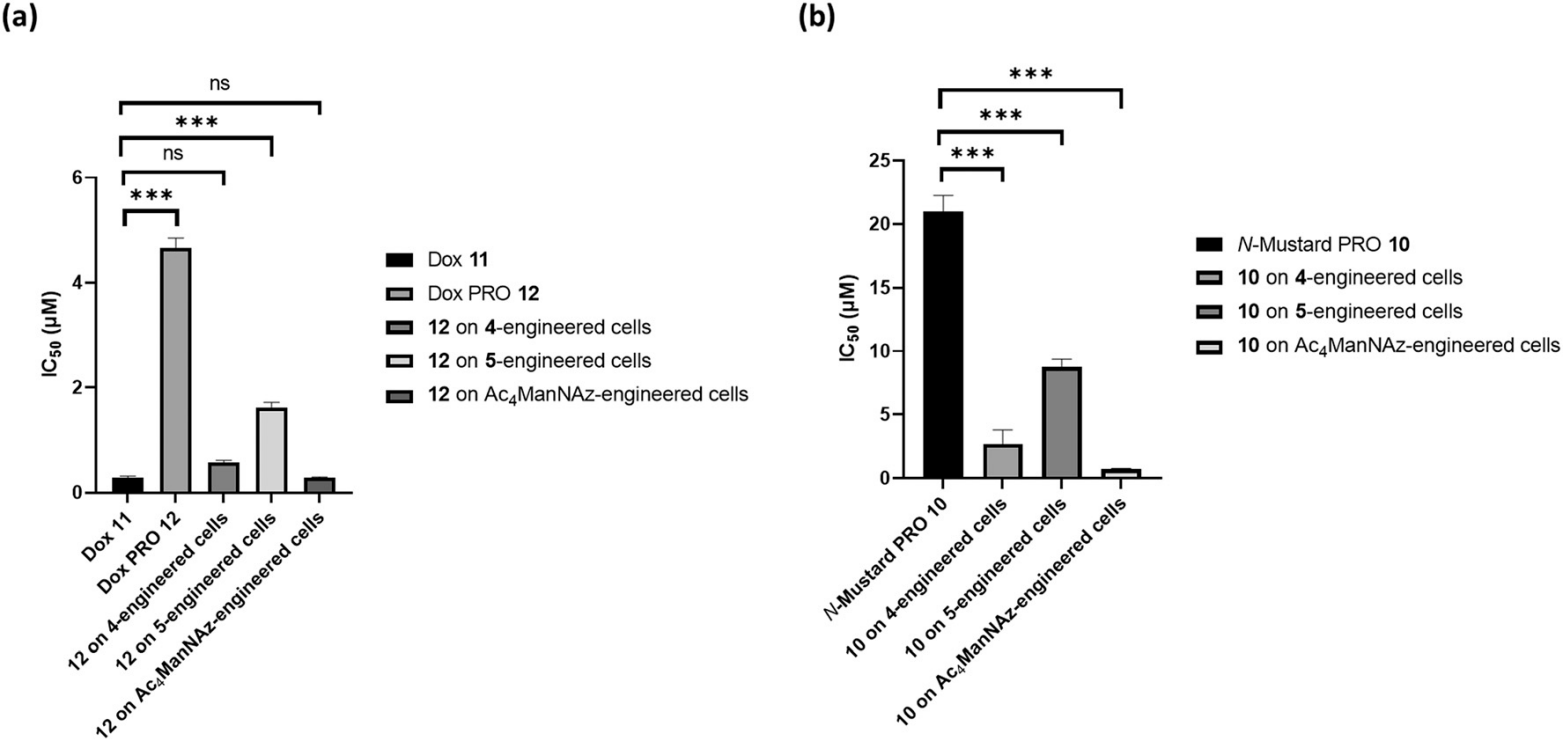
(a) Determined IC_50_ of Dox 11 and its prodrug 12 against MCF-7 and IC_50_ of prodrug 12 against 4, 5 and Ac_4_ManNAz-engineered MCF-7 cells. (b) Determined IC_50_ of prodrug 10 against MCF-7 and IC_50_ of prodrug 10 against 4, 5 and Ac_4_ManNAz-engineered MCF-7 cells. Data are presented as mean ± SEM (*n* = 3). ns represents no significance, *** indicates significance at *p* < 0.001.

First, the IC_50_ of the active doxorubicin drug 11 and the prodrug 12 were determined on MCF-7 cells that had not been previously treated with any azide-sugar derivatives and were found to be 0.2 μM and 4.6 μM, respectively. This demonstrates that forming a prodrug successfully masked doxorubicin's activity (more than 20-fold decrease in potency). Then, to test for prodrug activation, MCF-7 cells were first pre-treated with the different azide-modified sugars, Ac_4_ManNAz, 4 and 5, at a concentration of 50 μM for azide reporter expression, then cells were treated with the prodrug 12. The IC_50_ values (Table 1), (Fig. 5) and (Fig. S3a and b†) indicate successful prodrug activation with restoration of the active doxorubicin's IC_50_ in a pattern aligned with the azide incorporation level achieved by the three azide-modified sugars previously shown by Western blotting and confocal microscopy imaging. The similar protocol was applied with the N-mustard drug and prodrug 10, however, the IC_50_ of the active N-mustard drug could not be determined due to the high instability of the bis-chloro amino derivative which is the active cytotoxic moiety to be released from the prodrug. The IC_50_ for the N-mustard prodrug 10 was found to be 20.8 μM and after testing the prodrug activation as previously described with the doxorubicin prodrug, this IC_50_ value decreased as noticed with the doxorubicin prodrug 12 indicating the successful prodrug activation by the Staudinger reaction (Table 1) and (Fig. 5).

To further test the safety of the prodrugs 10 and 12 on normal cells, the IC_50_ values of doxorubicin prodrug 12 and N-mustard prodrug 10 were determined on L929 cells and were found to be 11.3 μM and 31.4 μM, respectively which are approximately 2 fold higher than for the MCF-7 cells. These results verify the better safety profile of the prodrugs 10 and 12 in normal cells compared to cancerous cells (Fig. S3c and d†). Prodrug activation was also tested on L929 cells that had first been treated with Ac_4_ManNAz, 4 and 5, at a concentration of 50 μM. This was in order to determine whether any azide incorporation would occur, that could lead to undesirable activation of the prodrugs in non-cancerous cell lines. No activation of prodrugs 10 and 12 was observed with the cells pre-treated with azido-sialic acid derivatives 4 and 5 (Table 1) indicating the validity of our hypothesis, specifically that azide incorporation occurs within the breast cancer cells but not within the non-cancerous L929 cells.

These results demonstrate that when doxorubicin and N-mustard cytotoxic moieties are combined with a triphenyl phosphine moiety forming a prodrug, the cytotoxic effect of the active drug is masked. MGE, azide expression and the bioorthogonal Staudinger reaction lead to their selective activation at breast cancer cells where their cytotoxic activity is unmasked and restored. This strategy increases both the selectivity and the safety profile for these drugs.

## 3. Conclusion

In summary, this study is the first to utilize a combined metabolic glycoengineering and Staudinger reaction approach for targeted prodrug activation at breast cancer cells to improve the tumor-targeting abilities of non-selective chemotherapeutics. A two-component bioorthogonal reaction system relying on the Staudinger reaction was developed and tested for its feasibility for selective prodrug activation *in vitro*. Two 9-azido sialic acid derivatives 4 and 5 were developed along with two novel phosphine-modified prodrugs 10 and 12 containing the N-mustard and doxorubicin cytotoxic moieties, respectively. Artificial azide-reporters were successfully generated onto MCF-7 breast cancer cells in a selective manner against normal L929 fibroblast cells (17-folds higher) indicating the selective tumor labelling approach. Excellent levels of potency restoration of the developed prodrugs were achieved on the azide-labelled cells indicating the feasibility of the labelling by MGE and activation by the Staudinger reaction.

This prodrug activation approach allows an advanced level of quantitative control over drug release as it relies on the level of the engineered azide on the tumor cells' surfaces. It also provides theoretical and experimental support for the design of MGE and bioorthogonal reactions-based targeted delivery systems for addressing the selectivity problems of conventional chemotherapies. Increasing the selectivity profiles of those chemotherapies can have a significant impact on their clinical usage, for example by increasing their selectivity will decreasing their systemic toxicity and thereby reducing their total required doses.

## 4. Materials and methods

### 4.1. Chemistry materials

Starting materials, reagents, and solvents were purchased from commercial sources and used without further purification. All the chemicals and solvents were purchased from Sigma Aldrich, UK, unless otherwise specified. *N*-Acetylneuraminic acid was purchased from Dextra, UK. Thin layer chromatography was performed on Merck TLC Silica gel 60 F254 aluminium backed plates. Compounds were purified by flash column chromatography using Silica gel 60 (particle size 40–63 μm) supplied by Sigma Aldrich. ^1^H NMR, ^13^C NMR and ^31^P NMR spectra were recorded in either deuterium oxide (D_2_O) or deuterated chloroform (CDCl_3_) or deuterated DMSO (DMSO-*d*_6_) using a Bruker DPX 400 (400 MHz) spectrometer. Coupling constants (*J*) were expressed in hertz (Hz), chemical shifts (*δ*) of NMR were reported in parts per million (ppm) units relative to an internal standard (tetramethylsilane (TMS)). Infrared spectra were recorded on a Perkin Elmer precisely Spectrum 100 FT-IR spectrometer. Mass spectrometry data were recorded on a Thermo Fisher LTQ Orbitrap XL instrument. Release reaction studies and the purities of the target compounds were measured by reverse-phase high-performance liquid chromatography (HPLC) Hewlett-Packed Series 1100 system, with an ACE C18 reverse phase column (250 × 4.6 mm, 5 μm particle size, 300 Å pore size). UV-vis spectrophotometers (Cary 300 Bio UV-visible spectrophotometer and Jenway-7315 spectrophotometer) were used to record ultraviolet absorbance of the target compounds.

### 4.2. Synthesis

#### 5-Acetamido-3,5-dideoxy-β-d-*glycero*-d-galacto-2-nonulosonic acid methyl ester (2)

*N*-Acetylneuraminic acid (Neu5Ac) 1 (1.00 g, 3.23 mmol) was dissolved in dry methanol (20 mL) and trifluoroacetic acid (0.07 ml, 11.64 mmol) was added. The mixture was stirred overnight at room temperature until the solution was clear. The solvent was removed under reduced pressure to yield Neu5Ac methyl ester 2 as a white solid (0.9 g, 86%); [*α*]^20^_D_ −18 (*c* 1.0, MeOH), lit.^52^ −31.7 (*c* 1.0, H_2_O); m.p. 182–184 C°, lit.^53^ 182–184 °C. ^**1**^**H NMR** (D_2_O, 400 MHz) *δ* 1.82 (1H, dd, *J* = 12.0 Hz, *J* = 13.0 Hz, H_3ax_), 1.95 (3H, s, NHCOC**H̲**_**3̲**_), 2.22 (1H, dd, *J* = 5.0 Hz, *J* = 13.0 Hz, H_3eq_), 3.45 (1H, dd, *J* = 5.0 Hz, *J* = 9.0 Hz, H_7_), 3.52 (1H, dd, *J* = 6.5 Hz, *J* = 12.0 Hz, H_9′_), 3.63 (1H, ddd, *J* = 2.5 Hz, *J* = 6.4 Hz, *J* = 9.0 Hz, H_8_), 3.71–3.77 (4H, m, COOC**H̲**_**3̲**_, H_9_), 3.83 (1H, t, *J* = 10.2 Hz, H_5_), 3.92–3.99 (2H, m, H_4_, H_6_). ^**13**^**C NMR** (D_2_O, 100 MHz) *δ* 22.02 (NHCO**C̲**H_3_), 38.62 (C_3_), 52.02 (C_5_), 53.45 (COO**C̲**H_3_), 63.11 (C_9_), 66.62 (C_4_), 68.16 (C_7_), 70.06 (C_8_), 70.30 (C_6_), 95.30 (C_2_), 171.37 (NH**C̲**OCH_3_), 174.80 (**C̲**OOCH_3_). **IR *ν***_**max**_ [cm^−1^] (powder) 3310 (O–H), 2954 (C–H), 1735 (C=O, ester), 1629 (C=O, amide), 1546 (NH–CO). ***m/z* (FTMS + ESI)** M^+^ (C_12_H_21_NO_9_) requires 323.1. Found 323.1.

#### 5-Acetamido-9-*O*-tosyl-3,5-dideoxy-β-d-*glycero*-d-galacto-2-nonulosonic acid methyl ester (3)

Neu5Ac methyl ester 2 (0.45 g, 1.39 mmol) was dissolved in pyridine (7 mL). The solution was cooled to 0 °C and *p*-toluenesulfonyl chloride (0.29 g, 1.53 mmol, 1.1 eq.) was added dropwise under an inert atmosphere. The mixture was left to warm to room temperature and then stirred overnight. Pyridine was removed under vacuum and the crude product was purified using flash column chromatography (ethyl acetate/methanol, 20 : 1) to yield the 5-acetamido-9-*O*-tosyl-3,5-dideoxy-β-d-*glycero*-d-galacto-2-nonulosonic acid methyl ester 3 as white solid (0.6 g, 90%);^54^ [*α*]^20^_D_ −16.4 (*c* 1.0, MeOH); m.p. 91–93 °C. ^**1**^**H NMR** (D_2_O, 400 MHz) *δ* 1.80 (1H, dt, *J* = 13.0 Hz, *J* = 11.6 Hz, H_3ax_), 1.97 (3H, s, NHCOC**H̲**_**3̲**_), 2.21 (1H, dd, *J* = 13.1 Hz, *J* = 4.9 Hz, H_3eq_), 2.38 (3H, s, C**H̲**_**3̲**_ (Ts)), 3.46–3.50 (1H, m, H_7_), 3.72–3.83 (5H, m, H_5_, COOC**H̲**_**3̲**_, H_8_), 3.91–4.16 (3H, m, H_6_, H_4_, H_9′_), 4.23 (1H, dd, *J* = 10.5 Hz, *J* = 2.4 Hz, H_9_), 7.43 (2H, d, *J* = 8.0 Hz ArH_3_/H_5_ (Ts)), 7.77 (2H, d, *J* = 8.5 Hz, ArH_2_/H_6_ (Ts)). ^**13**^**C NMR** (D_2_O, 100 MHz) *δ* 20.79 (CH_3_ (Ts)), 22.02 (NHCO**C̲**H_3_), 38.64 (C_3_), 52.00 (COO**C̲**H_3_), 53.47 (C_5_), 66.52 (C_4_), 67.52 (C_8_), 67.59 (C_7_), 70.09 (C_6_), 72.50 (C_9_), 95.26 (C_2_), 127.80 (C_2′_, C_6′_ (Ar)), 130.19 (C_3′_, C_5′_ (Ar)), 130.50 (C_4′_ (Ar)), 146.64 (C_1′_ (Ar)), 171.27 (NH**C̲**OCH_3_), 174.89 (**C̲**OOCH_3_). **IR *ν***_**max**_ [cm^−1^] (powder) 3354 (O–H), 3115 (C=C–H), 2946 (C–H), 1751 (C=O, ester), 1616 (C=O, amide), 1583 (NH–CO). ***m/z* (FTMS + ESI)** M^+^ (C_19_H_28_NO_11_S) requires 478.1. Found 478.1.

#### 9-Azido-5-acetamido-3,5,9-trideoxy-β-d-*glycero*-d-galacto-2-nonulosonic acid (4)

5-Acetamido-9-*O*-tosyl-3,5-dideoxy-β-d-*glycero*-d-galacto-2-nonulosonic acid methyl ester 3 (0.6 g, 1.26 mmol) and sodium azide (0.32, 4.9 mmol) were dissolved in water (5 mL) and then acetone was added (15 mL). The mixture was heated under reflux overnight. The solvents were removed under vacuum and the crude product was purified using flash column chromatography (dichloromethane/methanol, 10 : 1) to yield the 9-azido-5-acetamido-3,5,9-trideoxy-β-d-*glycero*-d-galacto-2-nonulosonic acid 4 as a pale yellow solid (0.19 g, 46%);^54^ [*α*]^20^_D_ −7.6 (*c* 1.1, MeOH); m.p. 118–119 °C. ^**1**^**H NMR** (D_2_O, 400 MHz) *δ* 1.74 (1H, dd, *J* = 12.8 Hz, *J* = 11.5 Hz, H_3ax_), 2.01 (3H, s, NHOC**H̲**_**3̲**_), 2.13 (1H, dd, *J* = 12.9 Hz, *J* = 4.8 Hz, H_3eq_), 3.27–3.42 (3H, m, H_7_, H_9_/H_9′_), 3.72–3.99 (4H, m, H_8_, H_6_, H_5_, H_4_). ^**13**^**C NMR** (D_2_O, 100 MHz) *δ* 22.06 (NHCO**C̲**H_3_), 39.35 (C_3_), 48.84 (C_6_), 52.24 (C_9_), 63.26 (C_4_), 67.28 (C_7_), 68.54 (C_8_), 70.27 (C_5_), 96.37 (C_2_), 154.43 (NH**C̲**OCH_3_), 174.72 (**C̲**OOH). **IR *ν***_**max**_ [cm^−1^] (powder) 3262 (O–H), 2040 (N_3_), 1614 (C=O). ***m/z* (FTMS + ESI)** (M–H)^−^ (C_11_H_17_N_4_O_8_) calcd. 333.1. Found 333.1. HPLC analysis: MeCN–H_2_O (70 : 30), 99.98% purity.

#### 9-Azido-5-acetamido-2,4,7,8-tetracetyl-3,5,9-trideoxy-β-d-*glycero*-d-galacto-2-nonulosonic acid (5)

9-Azido sialic acid 4 (50 mg, 0.2 mmol) was dissolved in 4 mL of pyridine. The solution was cooled to 0 °C in ice, and acetic anhydride (2 mL, 22 mmol) and a catalytic amount of DMAP were added. The reaction was then left to warm to room temperature and stirred overnight. The solvents were removed under vacuum and the crude product was purified using flash column chromatography (hexane/ethyl acetate, 10 : 1; hexane/ethyl acetate, 1 : 1) to yield the 9-azido-5-acetamido-2,4,7,8-tetracetyl-3,5,9-trideoxy-β-d-*glycero*-d-galacto-2-nonulosonic acid 5 as orange crystals (26 mg, 35%); [*α*]^20^_D_ −60.1 (*c* 1.0, MeOH); m.p. 124–126 °C. ^**1**^**H NMR** (D_2_O, 400 MHz) *δ* 1.79 (1H, dd, *J* = 13.4 Hz, *J* = 11.5 Hz, H_3ax_), 1.87 (6H, d, *J* = 2.5 Hz, OAc), 1.97 (3H, s, NHOC**H̲**_**3̲**_), 2.00 (3H, d, *J* = 2.5 Hz, OAc), 2.09 (3H, d, *J* = 4.2 Hz, OAc), 2.37 (1H, dd, *J* = 11.8 Hz, *J* = 4.8 Hz, H_3eq_), 3.85 (1H, t, *J* = 10.4 Hz, H_7_), 3.95–4.21 (2H, m, H_9_/H_9′_), 4.37 (1H, dd, *J* = 12.8 Hz, *J* = 2.7 Hz, H_8_), 5.14–5.03 (1H, m, H_5_), 5.21 (1H, dt, *J* = 11.1 Hz, *J* = 5.1 Hz, H_4_), 5.36 (1H, dd, *J* = 8.0 Hz, *J* = 1.8 Hz, H_6_). ^**13**^**C NMR** (D_2_O, 100 MHz) *δ* 20.10 (CO**C̲**H_3_), 20.25 (CO**C̲**H_3_), 20.43 (CO**C̲**H_3_), 21.80 (CO**C̲**H_3_), 22.55 (NHCO**C̲**H_3_), 36.54 (C_3_), 48.97 (C_6_), 61.47 (C_9_), 67.43 (C_4_), 69.38 (C_7_), 69.58 (C_8_), 70.72 (C_5_), 98.97 (C_2_), 171.05 (**C̲**OCH_3_), 172.41 (**C̲**OCH_3_), 173.28 (**C̲**OCH_3_), 173.80 (**C̲**OCH_3_), 174.30 (NH**C̲**OCH_3_), 180.32 (**C̲**OOH). **IR *ν***_**max**_ [cm^−1^] (powder) 3340 (O–H), 1751 (C=O, ester), 1616 (C=O, amide). ***m/z* (FTMS + ESI)** (M–H)^−^ (C_19_H_25_N_4_O_12_) requires 501.4. Found 501.2. HPLC analysis: MeCN–H_2_O (70 : 30), 99.34% purity.

#### *N*,*N*-Bis-(2-hydroxyethyl)-4-nitroaniline (7)

To a solution of 1-chloro-4-nitrobenzene 6 (1 g, 6.3 mmol) in anhydrous DMSO (10 mL), was added diethanolamine (2.2 mL, 19 mmol). The reaction mixture was heated at reflux at 140 °C overnight. The hot reaction mixture solution was then poured on ice water and filtered to yield 7 as a yellow solid. Purification was carried out through recrystallization from hot hexane to afford the *N*,*N*-bis-(2-hydroxyethyl)-4-nitroaniline 7 as a yellow solid (0.99 g, 69%) m.p. 69 °C. ^**1**^**H NMR** (DMSO-*d*_6_, 400 MHz) *δ* 3.59 (8H, t, *J* = 5.8 Hz, C**H̲**_**2̲**_C**H̲**_**2̲**_OH), 4.83–4.88 (2H, m, OH), 6.82 (2H, d, *J* = 9.5 Hz, Ar–H), 8.02 (2H, d, *J* = 9.5 Hz, Ar–H). ^**13**^**C NMR** (DMSO-*d*_6_, 100 MHz) *δ* 53.72 (**C̲**H_2_CH_2_OH), 58.42 (CH_2_**C̲**H_2_OH), 111.21 (Ar–CH), 126.34 (Ar–CH), 135.69 (Ar–CH), 153.91 (Ar–CH). **IR *ν***_**max**_ [cm^−1^] (powder) 3350 (OH), 3103 (C=C–H), 2954 (C–H), 1601, 1576 (NO_2_ stretch). ***m/z* (FTMS + ESI)** M^+^ (C_10_H_14_N_2_O_4_) requires 226.1. Found 226.1.

#### *N*,*N*-Bis-(2-chloroethyl)-4-nitroaniline (8)

A solution of 7 (210 mg, 0.93 mmol) in pyridine was cooled to 0 °C and purged with nitrogen, then methanesulfonyl chloride (0.3 mL, 3.77 mmol) was added dropwise *via* a syringe under nitrogen for 0.5 hour. The reaction mixture was then left to warm to room temperature and heated at reflux at 80 °C for one hour. The solvent was removed under vacuum and the oily residue was partitioned between DCM/H_2_O (1 : 1100 mL). The organic layer was dried over anhydrous MgSO_4_, filtered and concentrated under vacuum. Purification was carried out through recrystallization from hexane to afford the *N*,*N*-bis-(2-chloroethyl)-4-nitroaniline 8 as orange crystals (143 mg, 59%). m.p. 87 °C. ^**1**^**H NMR** (DMSO-*d*_6_, 400 MHz) *δ* 3.81 (4H, t, *J* = 6.4 Hz, C**H̲**_**2̲**_CH_2_Cl), 3.90 (4H, t, *J* = 6.7 Hz, CH_2_C**H̲**_**2̲**_Cl), 6.99 (2H, d, *J* = 9.5 Hz, Ar–H), 8.12 (2H, d, *J* = 9.5 Hz, Ar–H). ^**13**^**C NMR** (CDCl_3_, 100 MHz) *δ* 39.90 (**C̲**H_2_CH_2_Cl), 53.41 (CH_2_**C̲**H_2_Cl), 110.76 (Ar–CH), 124.97 (Ar–CH), 126.45 (Ar–CH), 129.61 (Ar–CH). **IR *ν***_**max**_ [cm^−1^] (powder) 3011 (C=C–H), 2897.54 (C–H), 1582.68, 1476.96 (NO_2_ stretch). ***m/z* (FTMS + ESI)** M^+^ (C_10_H_12_N_2_O_2_Cl_2_) requires 262.03. Found 262.01.

#### *N*,*N*-Bis-(2-chloroethyl)benzene-1,4-diamine dihydrochloride salt (9)

To a solution of 8 (0.33 g, 1.25 mmol) in DCM : MeOH (1 : 1), RANEY® Ni (2 ml, water slurry) and hydrazine monohydrate (0.05 ml, 1.5 mmol) were added. The reaction mixture was stirred at room temperature for 4 hours. The reaction mixture was filtered on Celite and washed with DCM. After filtration and drying over anhydrous MgSO_4_, the solvent was concentrated under vacuum to dryness. The crude product was then immediately re-dissolved in dry HCl in ether (1 ml) to form the dihydrochloride salt 9 as white powder (0.2 g, 68%). m.p. 207 °C (charring). ^**1**^**H NMR** (DMSO-*d*_6_, 400 MHz) *δ* 3.63–3.75 (8H, m, C**H̲**_**2̲**_C**H̲**_**2̲**_Cl), 6.77 (2H, d, *J* = 9.0 Hz, Ar–H), 7.03 (2H, d, *J* = 8.1 Hz, Ar–H). ^**13**^**C NMR** (DMSO-*d*_6_, 100 MHz) *δ* 47.40 (**C̲**H_2_CH_2_Cl), 53.33 (CH_2_**C̲**H_2_Cl), 116.81 (Ar–CH), 123.80 (Ar–CH), 124.28 (Ar–CH), 129.92 (Ar–CH). ***m/z* (FTMS + ESI)** M^+^ (C_10_H_14_N_2_Cl_2_) requires 233.06. Found 233.06.

#### *N*-(4-(Bis(2-chloroethyl)amino)phenyl)-2-(diphenylphosphaneyl)benzamide (10)

2-Diphenylphosphanyl benzoic acid (0.2 g, 0.62 mmol) was dissolved in 20 mL of anhydrous DCM under an inert atmosphere. DCC (0.14 g, 0.68 mmol) and DMAP (0.004 g, 0.03 mmol) were added to the reaction mixture which was kept under an inert atmosphere. The reaction mixture was stirred at room temperature for 30 minutes and then the dihydrochloride salt 9 (0.14 g, 0.62 mmol) was added with TEA (0.1 ml). The mixture was stirred overnight at room temperature. The solvent was removed under vacuum and ice-cold acetone was added to the residue to precipitate the urea by-product to be removed by filtration. After removal of acetone under vacuum, the crude product was purified using flash column chromatography (hexane/ethyl acetate, 7 : 3) to yield the *N*-(4-(bis(2-chloroethyl)amino)phenyl)-2-(diphenylphosphaneyl)benzamide 10 as pale yellow solid (97 mg, 31%). m.p. 211 °C. ^**1**^**H NMR** (DMSO-*d*_6_, 400 MHz) *δ* 3.71 (8H, m, C**H̲**_**2̲**_C**H̲**_**2̲**_Cl), 6.70 (2H, d, *J* = 9.1 Hz, Ar–H), 6.97 (1H, dd, *J* = 6.8, 3.8 Hz, Ar–H), 7.14–7.26 (4H, m, Ar–H), 7.36 (6H, m, Ar–H), 7.42 (2H, d, *J* = 9.2 Hz, Ar–H), 7.51 (1H, t, *J* = 7.0 Hz, Ar–H), 7.67 (1H, dd, *J* = 6.6, 3.7 Hz, Ar–H), 10.13 (1H, s, NH). ^**13**^**C NMR** (DMSO-*d*_6_, 100 MHz) *δ* 41.69 (**C̲**H_2_CH_2_Cl), 52.75 (CH_2_**C̲**H_2_Cl), 112.42 (Ar–CH), 122.25 (Ar–CH), 128.91 (Ar–CH), 128.98 (Ar–CH), 129.25 (Ar–CH), 133.61 (Ar–CH), 133.81 (Ar–CH), 134.40 (Ar–CH), 138.26 (Ar–CH), 138.38 (Ar–CH), 143.25 (C=O). ^**31**^**P NMR** (DMSO-*d*_6_, 162 MHz) *δ* −10.89. ***m/z* (FTMS + ESI)** M^+^ (C_29_H_27_N_2_Cl_2_OP) requires 519.1. Found 520.9. HPLC analysis: MeCN–H_2_O (70 : 30), 98.16% purity.

#### 2-(Diphenylphosphino)-*N*-((2*R*,3*R*,4*R*)-3-hydroxy-2-methyl-6-(((1*S*,3*S*)-3,5,12-trihydroxy-3-(2-hydroxyacetyl)-10-methoxy-6,11-dioxo-1,2,3,4,6,11-hexahydrotetracen-1-yl)oxy)tetrahydro-2*H*-pyran-4-yl)benzamide (12)

2-Diphenylphosphanyl benzoic acid (5.8 mg, 0.02 mmol) was dissolved in 5 mL of anhydrous DCM under an inert atmosphere. DCC (4.1 mg, 0.02 mmol) and DMAP (1 mg, 0.007 mmol) were added to the reaction mixture which was kept under an inert atmosphere. The reaction mixture was stirred at room temperature for 30 minutes and then doxorubicin 11 (10 mg, 0.02 mmol) was added. The mixture was stirred overnight at room temperature. The solvent was removed under vacuum and ice-cold acetone was added to the residue to precipitate the urea by-product to be removed by filtration. After removal of acetone under vacuum, the crude product was purified using flash column chromatography (hexane/ethyl acetate, 7 : 3) to yield the 2-(diphenylphosphaneyl)-*N*-(3-hydroxy-2-methyl-6-(((1*S*,4*R*)-4,5,12-trihydroxy-4-(2-hydroxyacetyl)-10-methoxy-6,11-dioxo-1,2,3,4,6,11-hexahydrotetracen-1-yl)oxy)tetrahydro-2*H*-pyran-4-yl)benzamide 12 as red fine powder (6 mg, 39%). m.p. 253 °C (charring). ^**1**^**H NMR** (DMSO-*d*_6_, 700 MHz) *δ* 0.98–1.07 (2H, m), 1.07–1.16 (2H, m), 1.17–1.31 (3H, m, C**H̲**_**3̲**_), 1.44–1.55 (1H, m), 1.59 (2H, ddd, *J* = 23.2, 13.5, 9.4 Hz), 1.62–1.66 (1H, m), 1.66–1.73 (1H, m), 1.78 (1H, ddd, *J* = 36.1, 18.8, 7.7 Hz), 1.89–2.04 (1H, m), 3.06–3.23 (1H, m), 3.43–3.54 (1H, m), 3.87 (3H, s, OC**H̲**_**3̲**_), 5.65 (1H, d, *J* = 7.9 Hz), 6.58 (1H, dd, *J* = 7.4, 3.2 Hz), 6.99–7.68 (16H, m, Ar–H), 7.89–7.97 (1H, m, DOX Ar–H). ^**13**^**C NMR** (DMSO-*d*_6_, 176 MHz) *δ* 24.45, 24.66, 24.93, 25.38, 25.76, 25.97, 30.82, 31.86, 33.81, 34.95, 40.47, 48.01, 49.65, 55.11, 70.23, 127.83, 127.88, 128.35, 128.38, 128.49, 128.87, 128.90, 130.76, 131.30, 133.09, 133.29, 133.40, 133.67, 133.78, 137.59, 137.70, 142.31, 142.40, 153.30, 157.13, 168.70, 170.69. ^**31**^**P NMR** (DMSO, 162 MHz) *δ* −8.82. ***m/z* (FTMS + ESI)** M^+^ (C_46_H_42_NO_12_P) requires 831.8. Found 832.2. HPLC analysis: MeCN–H_2_O (70 : 30), 95.44% purity.

### 4.3. HPLC release study

Doxorubicin prodrug 12 (0.0415 mg mL^−1^) (50 μM) or N-mustard prodrug 10 (0.026 mg mL^−1^) (50 μM) in 2 mL of CH_3_CN/H_2_O (1 : 1) was reacted with the 9-azido sialic acid 4 trigger (0.0334 mg mL^−1^) (100 μM) at 37 °C and at different time intervals, samples of 25 μL were withdrawn and analyzed by HPLC. The flow rate was 1 mL min^−1^, the mobile phase was 70% acetonitrile and 30% water. The injection volume was 25 μL and the run time was 25 min, (UV detector at *λ* = 233 nm for 12 and *λ* = 254 nm for 10).

### 4.4. Cell culture materials

Human breast adenocarcinoma epithelial cell line (MCF-7) and mouse fibroblasts (L929) were purchased from the American Type Culture Collection (ATCC, Rockville, MD, USA). For cell culture, cell culture medium RPMI 1640 (with l-glutamine) and DMEM were purchased from Lonza, UK. The fetal bovine serum (FBS) and trypsin were purchased from Gibco, UK. RIPA buffer with 1% protease inhibitor cocktail and phosphine-PEG_3_-biotin were purchased from Thermo Fisher Scientific, UK, streptavidin-HRP was purchased from Cell Signalling Technology, UK.

### 4.5. *In vitro* azide-reporter generation analysis

For azide generation analysis in breast cancer cells and mouse fibroblasts, MCF-7 cells were cultured in 5% fetal bovine serum and RPMI 1640 medium and L929 cells were cultured in 10% fetal bovine serum and DMEM medium at 37 °C in a 5% CO_2_ incubator.

#### Western blotting

To analyse the engineered azide groups in glycoproteins *in vitro*, 4 × 10^4^ cell per mL of MCF-7 and 1 × 10^4^ cell per mL of L929 cells were seeded into 6-well plates and incubated with 50 μM Ac_4_ManNAz-, 4-, and 5-containing medium for 72 h at 37 °C CO_2_ incubator, respectively. Cells were then washed twice with ice cold PBS and lysed using ice cold RIPA buffer (Thermo Fisher Scientific, UK) with 1% protease inhibitor cocktail. Lysates were centrifuged at 12 000 rpm for 10 min at 4 °C to remove cell debris. The total protein of each sample was quantified by DC assay (Bio-rad DC Protein Assay Kit, Bio-rad, UK). The lysates were then incubated with 0.5 μM phosphine-PEG_3_-biotin (Thermo Fisher Scientific, UK) for 12 h at room temperature. The proteins from each sample were mixed with 1× sodium dodecyl sulfate (SDS) gel-loading buffer (125 mol L^−1^ Tris, pH 6.8, 5% glycerol, 2% SDS, 1% β-mercaptoethanol, and 0.006% bromophenol blue) and boiled for 5 min. Then, 20 μg of proteins was separated by 8% SDS – polyacrylamide gel electrophoresis and transferred onto PVDF membranes. The membranes were blocked for 1.5 h at room temperature in 0.5% bovine serum albumin (BSA) containing 1× TBST solution (10 mol L^−1^ Tris, pH 7.4, 100 mol L^−1^ NaCl, and 0.1% Tween 20). Then, the membranes were incubated with streptavidin-HRP (Cell Signalling Technology, UK) containing 1× TBST solution for overnight at 4 °C. Next day, the membranes were washed three times using 1× TBST and protein band was detected with an ECL system.

#### Confocal microscopy imaging

For fluorescence quantification, 4 × 10^4^ cell per mL of MCF-7 and 1 × 10^4^ cell per mL of L929 cells were seeded into a 35 mm covered glass-bottom dishes and incubated with 50 μM Ac_4_ManNAz-, 4-, and 5-containing medium for 72 h at 37 °C CO_2_ incubator, respectively. Then to visualize the azide groups, the sugar-containing medium was removed and the cells were washed twice with PBS and further incubated with 20 μM dibenzylcyclooctyne-conjugated Cy5 (DBCO-Cy5)-containing fresh medium for 1 h at 37 °C. Then, the cells were washed twice with PBS and fixed with formaldehyde fixative for 10 min in dark conditions. After fixation, the cells were washed twice with PBS and mounted with 4,6-diamidino-2-phenylindole (DAPI) (5 μg mL^−1^). The cells were observed using a confocal laser microscope (Nikon A1R Confocal with 405, 445, 488, 514, 561 and 640 nm lasers). The mean fluorescence intensity was analyzed using ImageJ software and the data were represented as absolute values.

### 4.6. *In vitro* prodrug activation and anticancer activity

The *in vitro* prodrug activation and anticancer activity of compounds 10 and 12 was determined using the MTT assay. MCF-7 cells or L929 cells were incubated with 50 μM Ac_4_ManNAz-, 4-, and 5-containing RPMI medium or 50 μM Ac_4_ManNAz-, 4-, and 5-containing DMEM medium, respectively for 72 h at 37 °C CO_2_ incubator as described above. Then, the engineered and non-engineered MCF-7 cells or L929 cells were seeded on 96-well plates (4 × 10^4^ cells per mL) or (1 × 10^5^ cells per mL), respectively and incubated at 37 °C for 24 h. Cells were then treated with a range of concentration of both prodrugs 10 and 12 (0.001–10 μM) and incubated for 72 h. After incubation, the treatments-containing medium was removed and 20 μL of 0.5 mg mL^−1^ MTT in PBS solution was added in each well for 4 h. The resulting formazan crystals were dissolved in 100 μL of DMSO after carefully removing the MTT solution. The absorbance was recorded at 570 nm by InfiniteF50 TECAN microplate reader. The cells without any treatment were used as the control. Assays were performed in three replicates, which the statistical mean and standard deviation were used to estimate the cell viability. IC_50_ (inhibitory concentration to induce 50% cell death) values were determined using GraphPad Prism 8.0.2 according to the fitted data.

### 4.7. Statistical analysis

Data were presented as mean ± standard error of mean. Statistical analysis was carried out (for Dox prodrug 12 on unengineered MCF-7 cells or L929 cells and Dox prodrug 12 on 4, 5 and Ac_4_ManNAz-engineered MCF-7 cells or L929 cells against the active Dox 11 on MCF-7 cells or L929 cells, and for N-mustard prodrug 10 on 4, 5 and Ac_4_ManNAz-engineered MCF-7 cells or L929 cells against the N-mustard prodrug 10 on unengineered MCF-7 cells or L929 cells) by one-way ANOVA followed by Dunnett's *post hoc* test using GraphPad Prism 8.0.2 software and statistical significance was set at *p* < 0.05 (specifically, * for *p* < 0.05; ** for *p* < 0.01; *** for *p* < 0.001; **** for *p* < 0.0001).

## Conflicts of interest

The authors declare that they have no known competing financial interests or personal relationships that could have appeared to influence the work reported in this paper.

## Supplementary Material

MD-014-D3MD00137G-s001
