# Supplementary material for: Bioorthogonal activation of prodrugs, for the potential treatment of breast cancer, using the Staudinger reaction
Source: RSC Med Chem. 2023 Jul 5;14(8):1537–48. doi: 10.1039/d3md00137g (PMC10429771; doi:10.1039/d3md00137g)
Supplement: MD-014-D3MD00137G-s001 [file MD-014-D3MD00137G-s001.pdf]

Electronic Supplementary Material (ESI) for RSC Medicinal Chemistry.
This journal is © The Royal Society of Chemistry 2023

Supporting Information

Bioorthogonal activation of prodrugs, for the potential treatment of breast cancer, using the Staudinger reaction

Madonna M. A. Mitry [a,b], Samuel Y Boateng [c], Francesca Greco [a*], Helen M.I. Osborn [a*]

[a] Reading School of Pharmacy, University of Reading, Whiteknights, Reading, RG6 6AD. UK

[b] Dept. of Pharmaceutical chemistry, Faculty of Pharmacy, Ain Shams University, Cairo 11566, Egypt

[c] School of Biological Sciences, University of Reading, Whiteknights, Reading RG6 6UB, UK

*Corresponding author, E-mail: h.m.i.osborn@reading.ac.uk , f.greco@reading.ac.uk

Table of contents

Chromatograms of 9-azido sialic acid **4** with DOX prodrug **12** and *N*-Mustard prodrug **10** …………………………………………………………………………………………………… **2**

Viability of MCF-7 cells and L929 cells after treatment with $Ac_4ManNAz$, **4** or **5** ……………. **2**

IC_{50} of DOX prodrug **12** and *N*-Mustard prodrug **10** against MCF-7 cells and L929 cells….. **3**

Confocal microscopy images of untreated, $Ac_4ManNAz$, **4** and **5**-treated MCF-7 cells…….. **4**

^{1}H, ^{13}C and ^{31}P NMR spectra………………………………………………………………………. **6**

HPLC traces analysis of compounds **4**, **5**, **10** and **12**……………………………………………… **17**

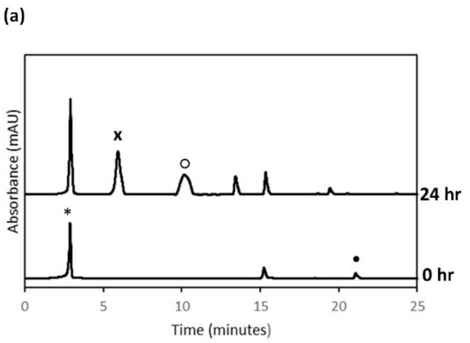

Figure *S1*: a) HPLC chromatograph of doxorubicin **11** release from prodrug **12** by 9-azido sialic acid **4** at 37 C° in aqueous MeCN (1:1) as a function of time. b) HPLC chromatogram of *N*-Mustard release from prodrug **10** by 9-azido sialic acid **4** at 37 C° in aqueous MeCN (1:1) as a function of time. Legend: *: azido-sialic acid **4**; •: Dox prodrug **12**; ○: Dox **11**; **x**: phosphine-oxide ligation product; ◊: *N*-Mustard prodrug **10**.

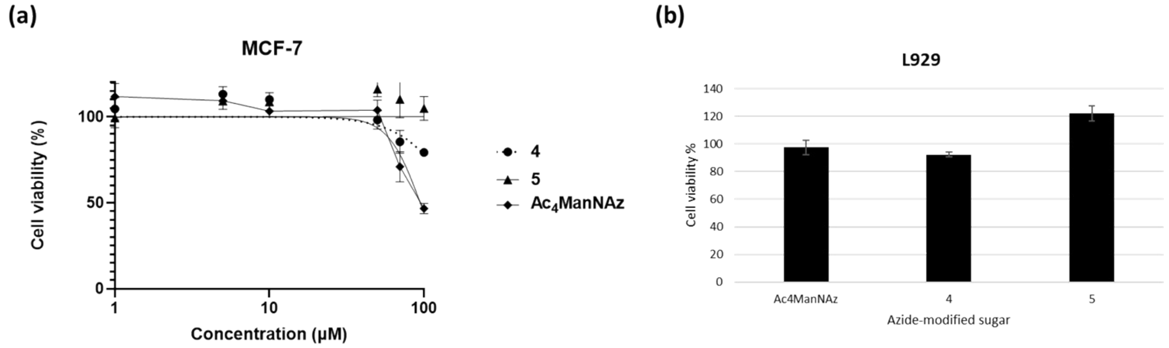

Figure *S2*: a) Viability of MCF-7 cells after incubation with $Ac_4ManNAz$, **4** or **5** at various concentrations (1-100 µM) determined by the MTT assay. b) Viability of L929 cells after incubation with 50 µM $Ac_4ManNAz$, **4** or **5** determined by the MTT assay. Data are presented as mean ± SEM (n = 3).

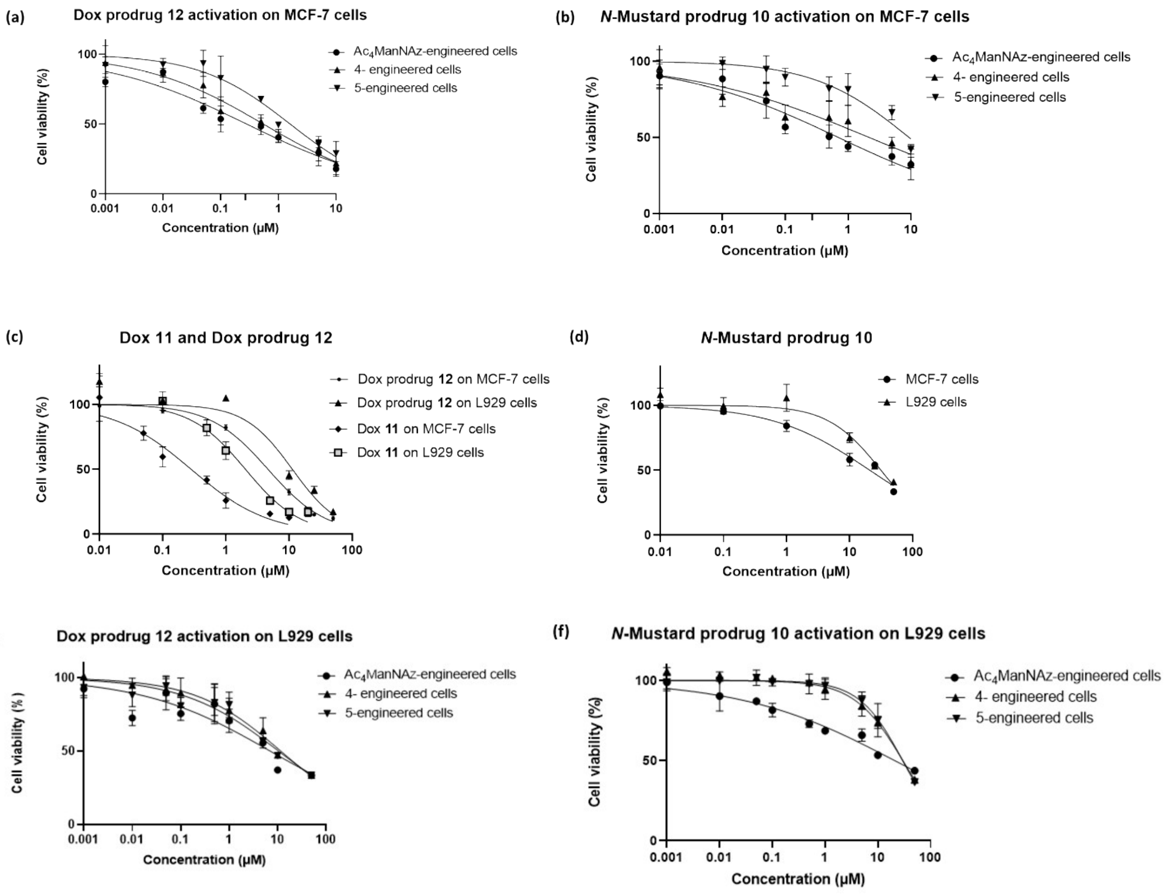

Figure *S*3: a) Determined IC_{50} for Dox prodrug **12** activation against **4**, **5** and Ac_4ManNAz-engineered MCF-7 cells. b) Determined IC_{50} for *N*-Mustard prodrug **10** activation against **4**, **5** and Ac_4ManNAz-engineered MCF-7 cells. c) Determined IC_{50} of Dox **11** and Dox prodrug **12** against MCF-7 cells and L929 cells. d) Determined IC_{50} of *N*-Mustard prodrug **10** against MCF-7 cells and L929 cells. e) Determined IC_{50} for Dox prodrug **12** activation against **4**, **5** and Ac_4ManNAz-engineered L929 cells. f) Determined IC_{50} for *N*-Mustard prodrug **10** activation against **4**, **5** and Ac_4ManNAz-engineered L929 cells. Data are presented as mean ± SEM (n=3).

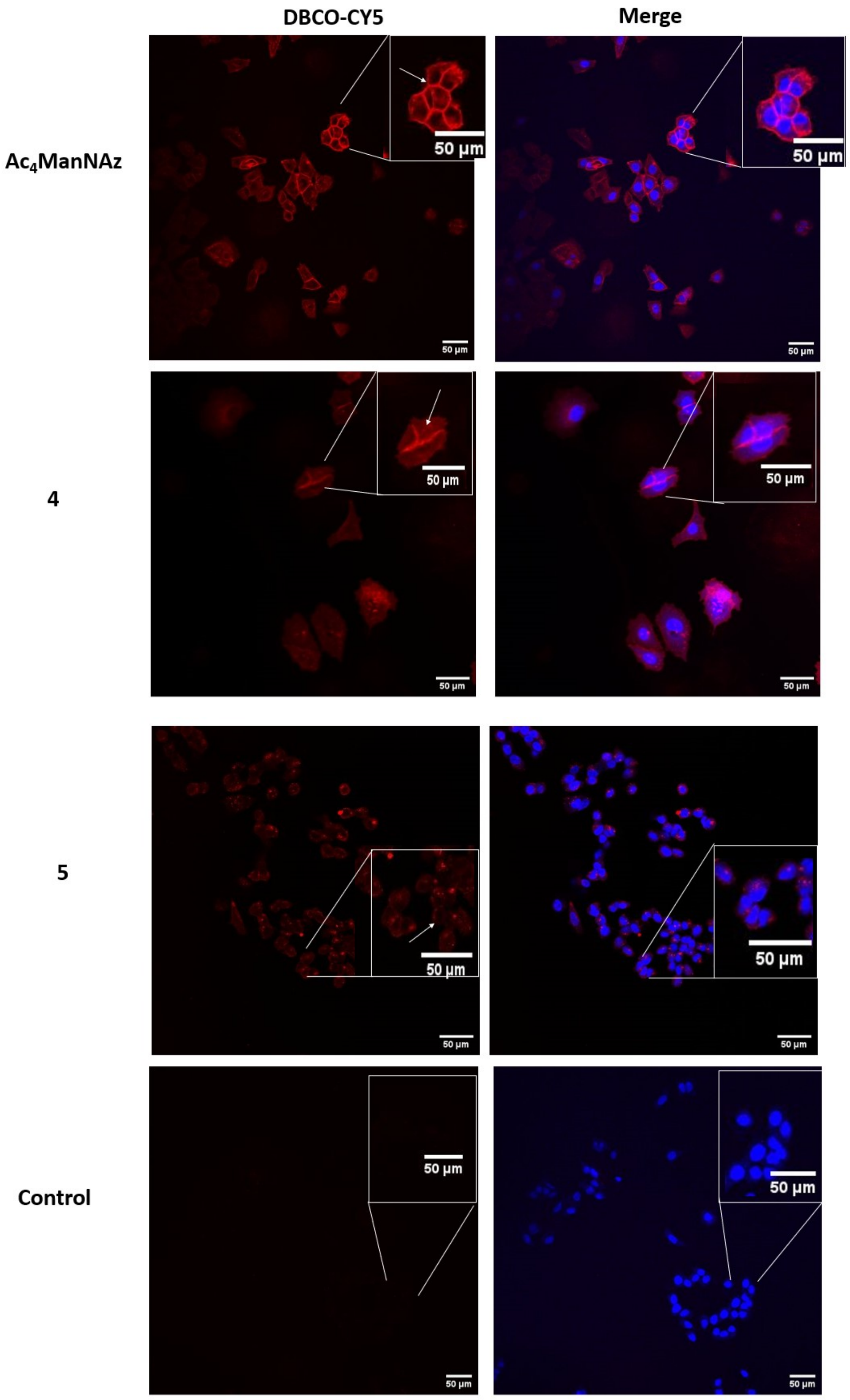
DBCO-CY5
Merge
Ac4ManNAz
4
5
Control
50 µm

Figure *S*4: Confocal fluorescence microscopy images of Ac_4ManNAz, **4**, **5**-treated and untreated control breast cancer cells (MCF-7) (50 µM for 72 h). Arrows indicate higher fluorescence at cell membranes and cell membrane junctions between cells.

^{1}H and ^{13}C NMR Spectra

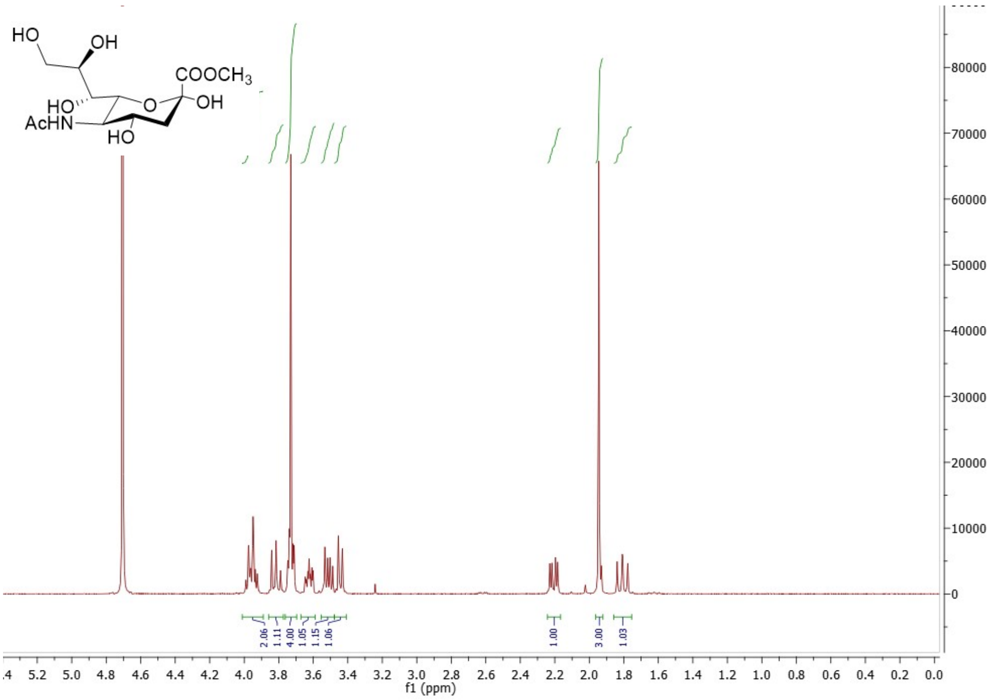

^{1}H NMR spectrum (400 MHz, D_2O) of compound **2**

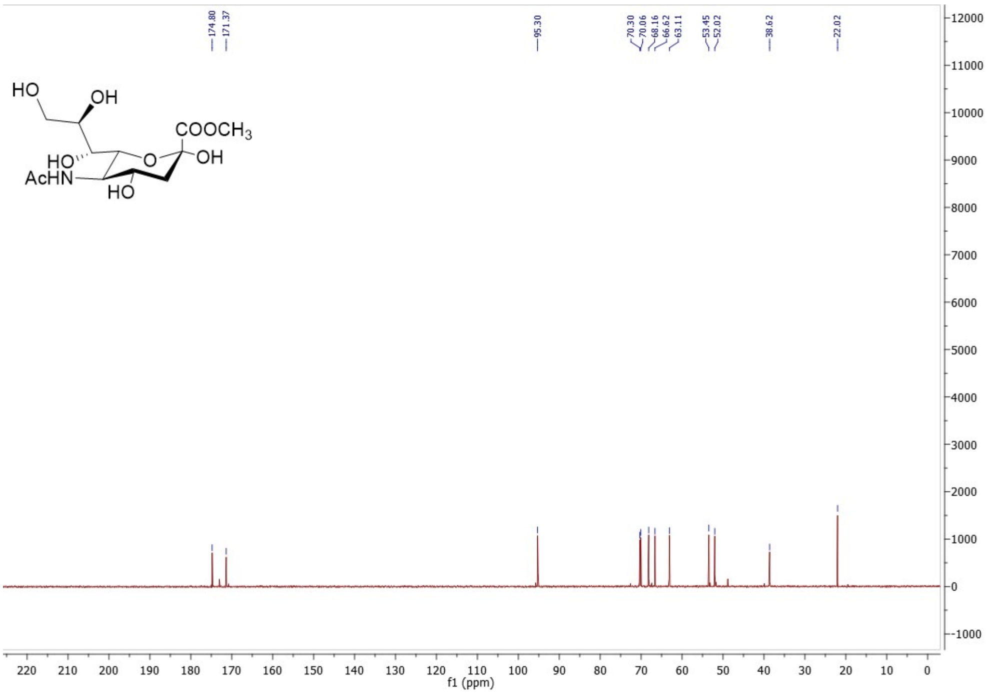

^{13}C NMR spectrum (100 MHz, D_2O) of compound **2**

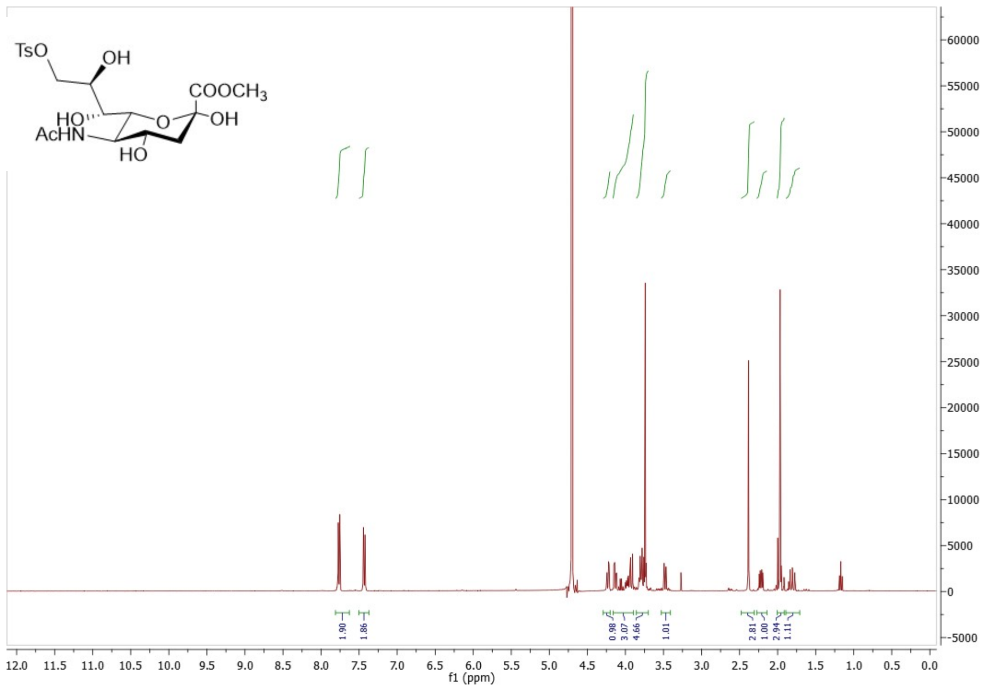

^{1}H NMR spectrum (400 MHz, D_2O) of compound **3**

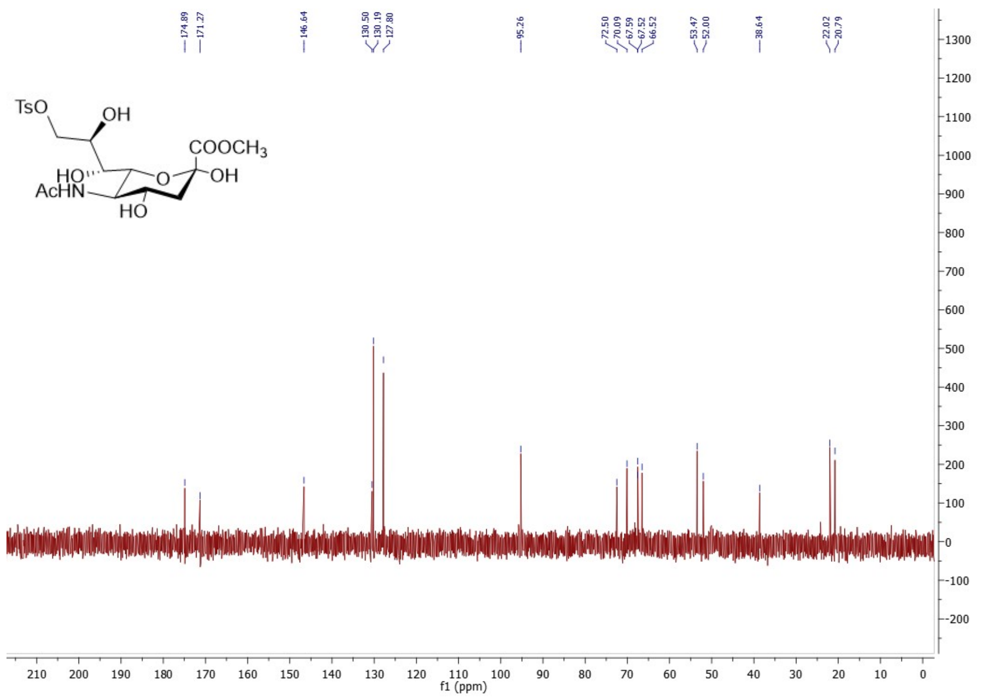

^{13}C NMR spectrum (100 MHz, D_2O) of compound **3**

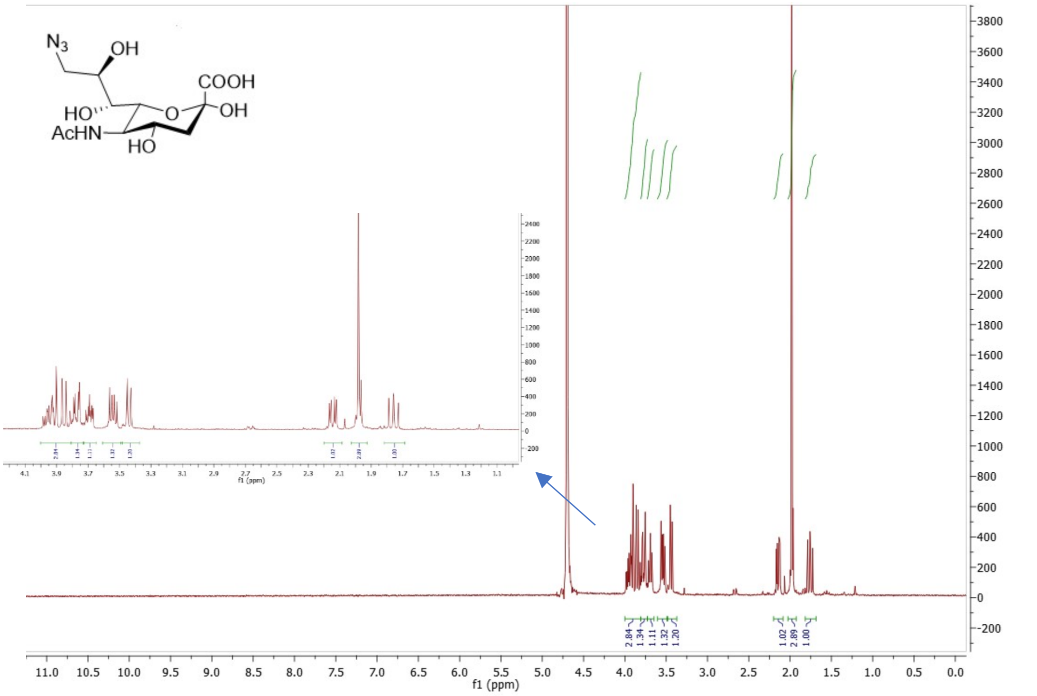

^{1}H NMR spectrum (400 MHz, D_2O) of compound **4**

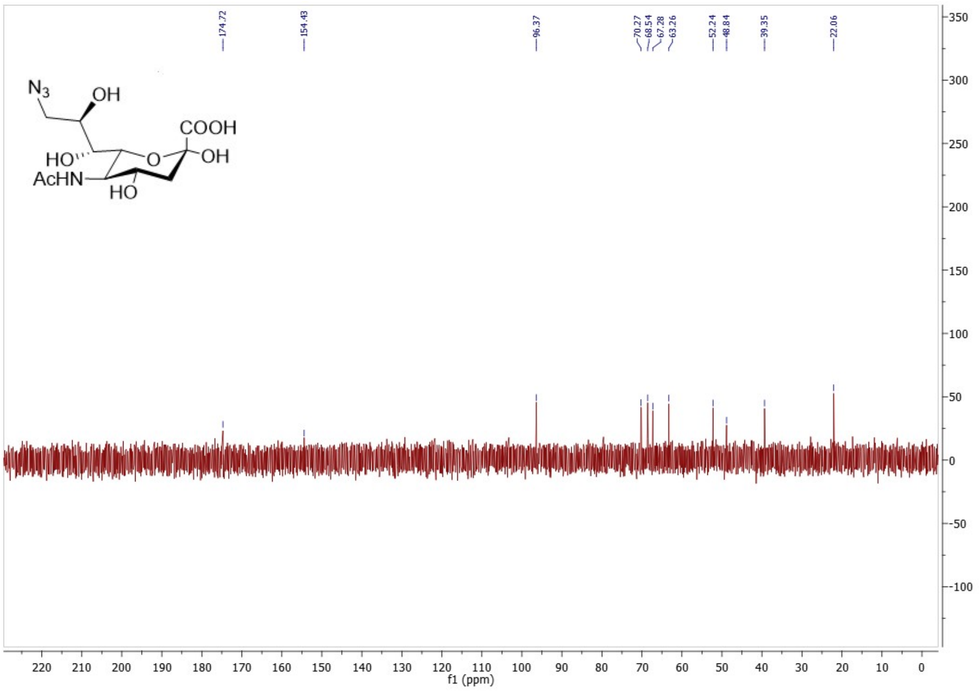

^{13}C NMR spectrum (100 MHz, D_2O) of compound **4**

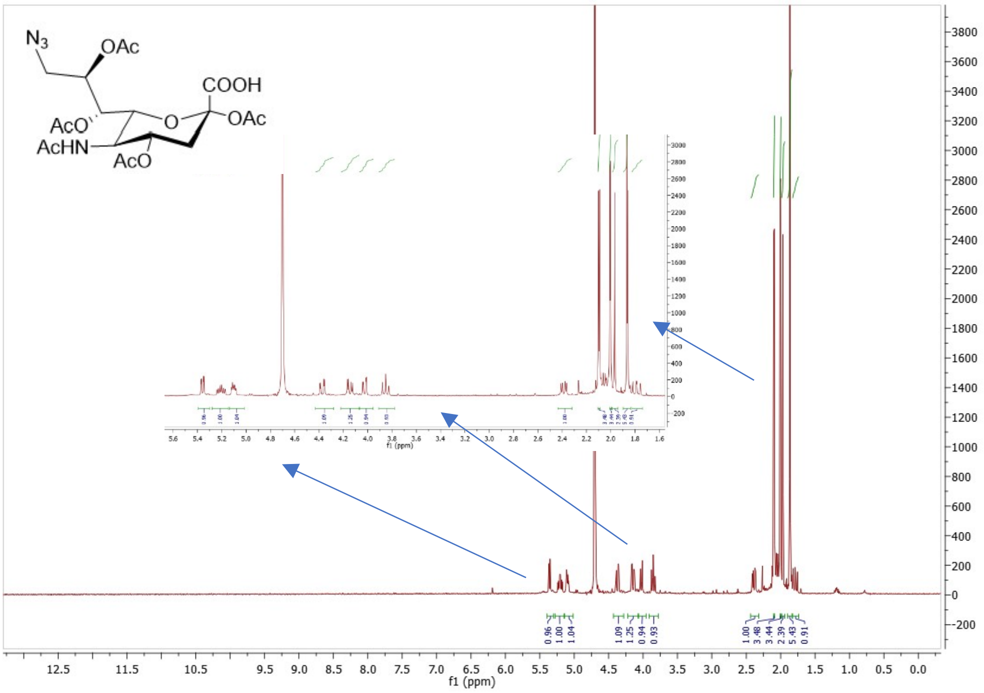

^{1}H NMR spectrum (400 MHz, D_2O) of compound **5**

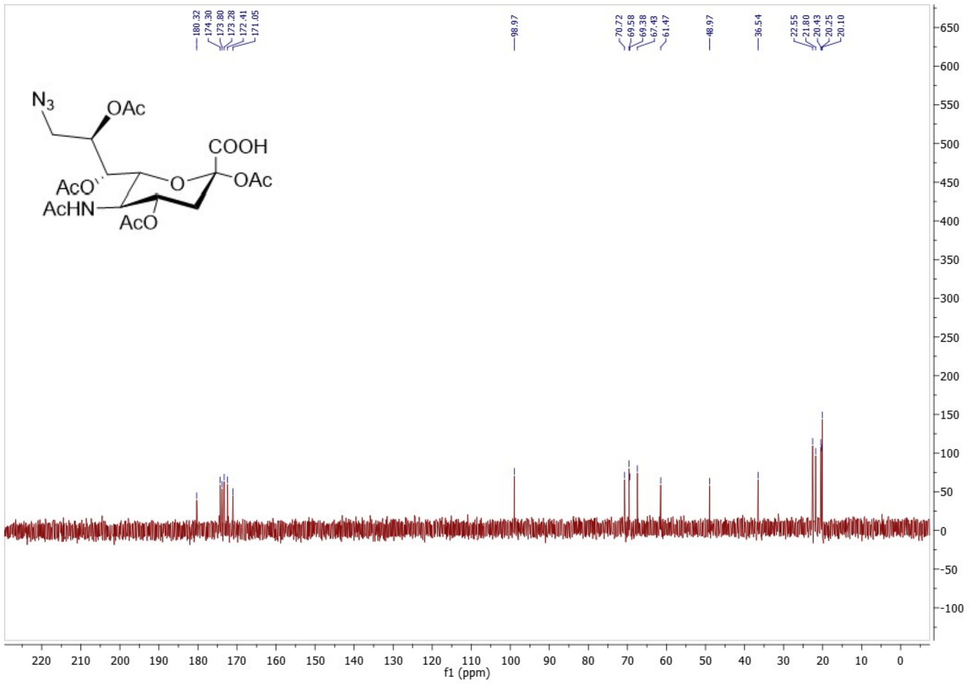

^{13}C NMR spectrum (100 MHz, D_2O) of compound **5**

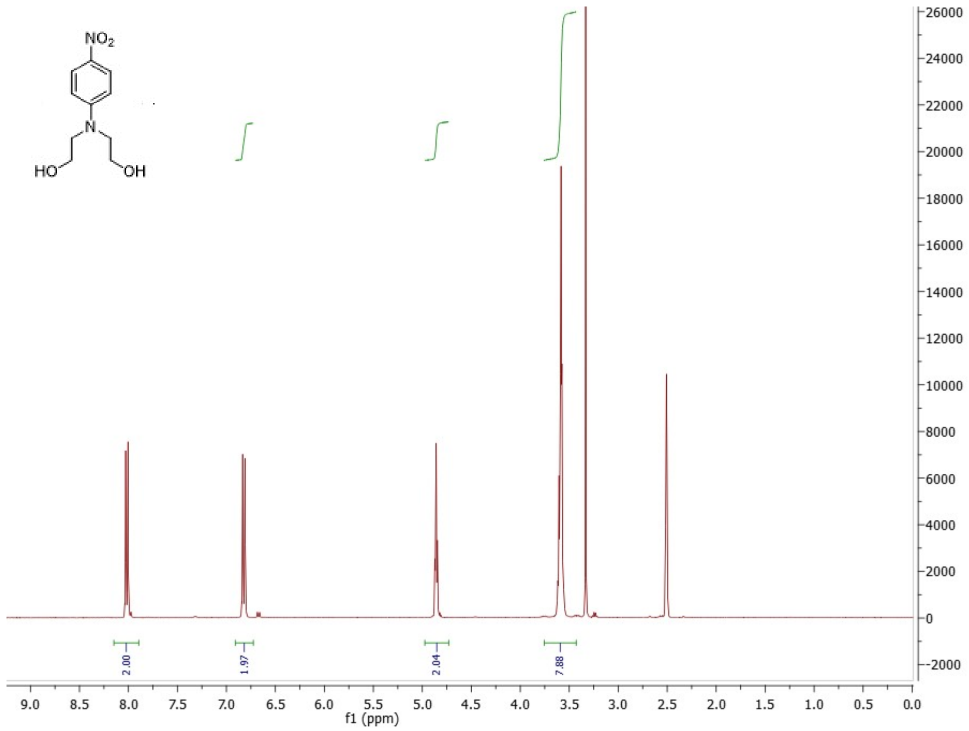

^{1}H NMR spectrum (400 MHz, DMSO-d_6) of compound **7**

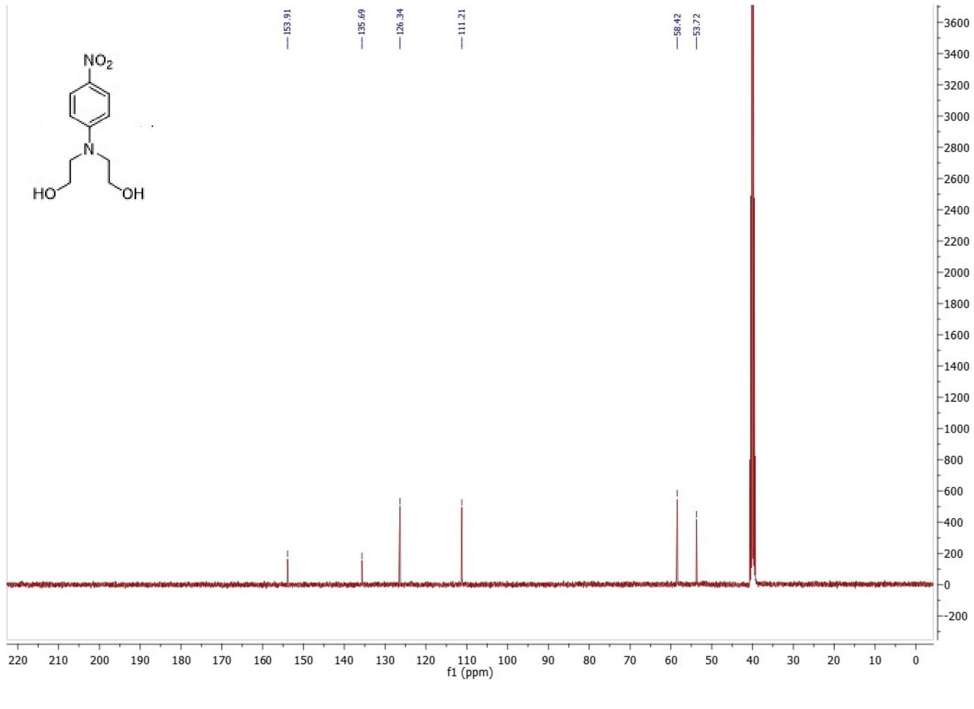

^{13}C NMR spectrum (100 MHz, DMSO-d_6) of compound **7**

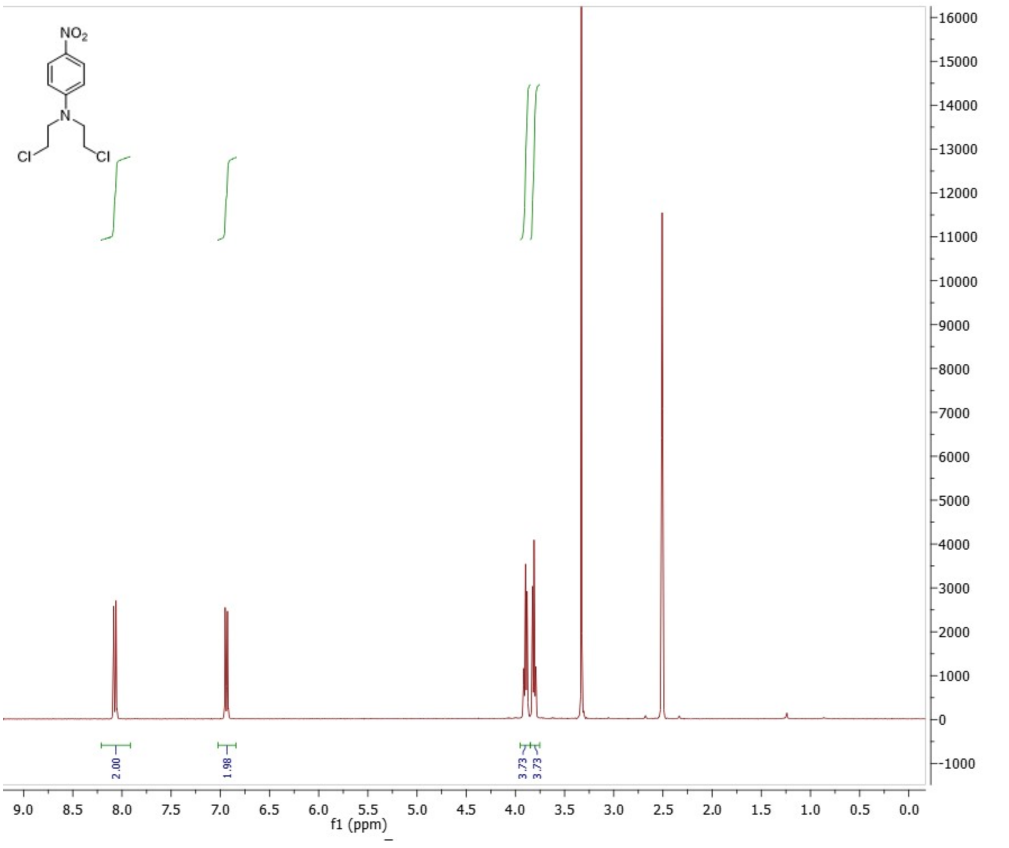

^{1}H NMR spectrum (400 MHz, DMSO-d_6) of compound **8**

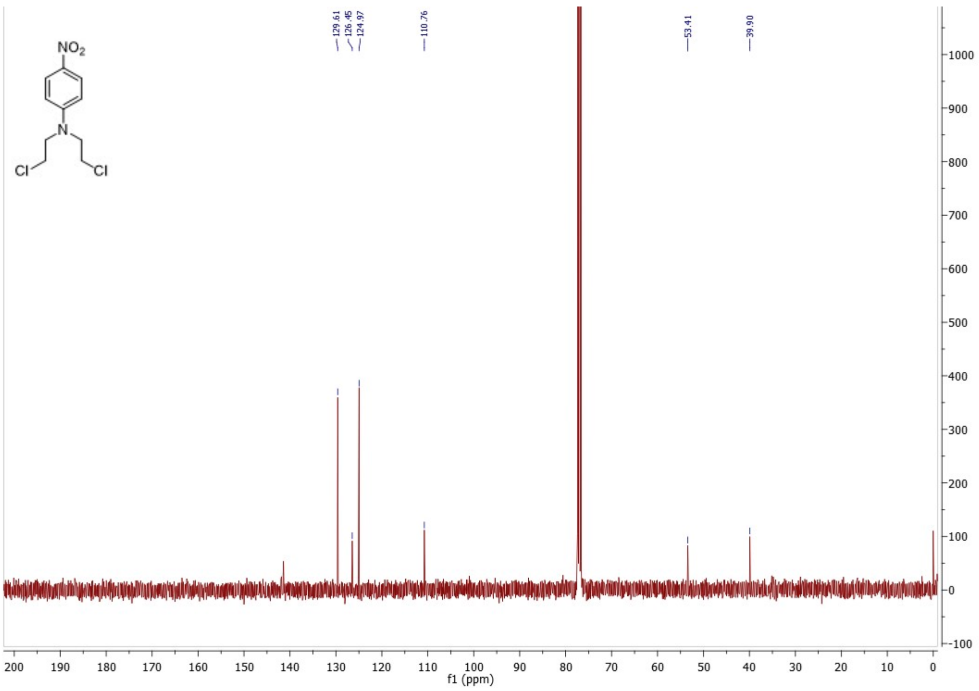

^{13}C NMR spectrum (100 MHz, $CDCl_3$) of compound **8**

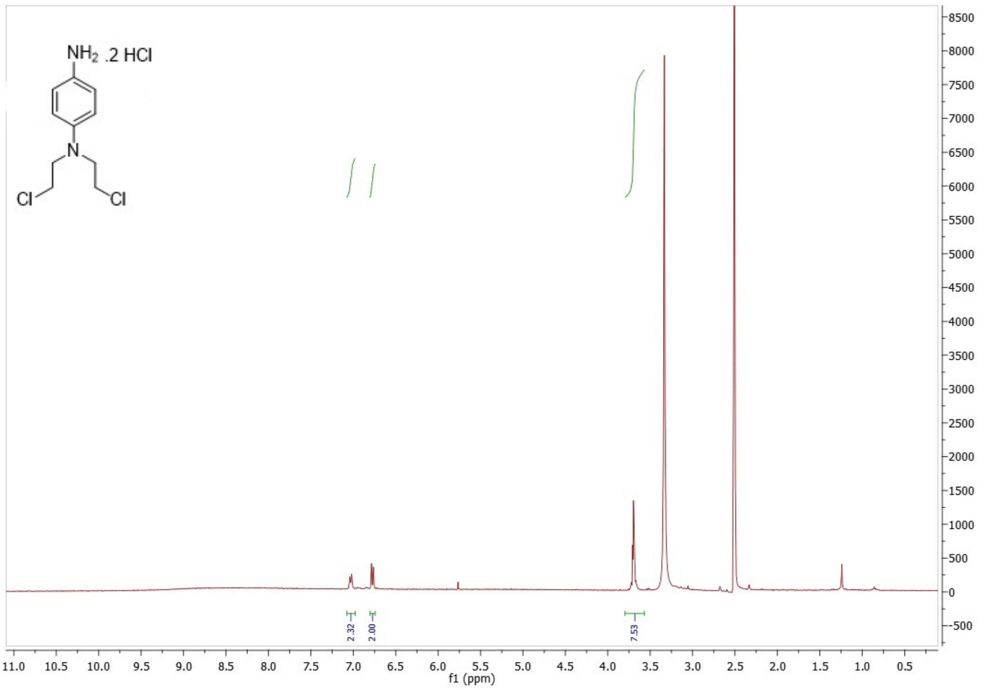

^{1}H NMR spectrum (400 MHz, DMSO-d_6) of compound **9**

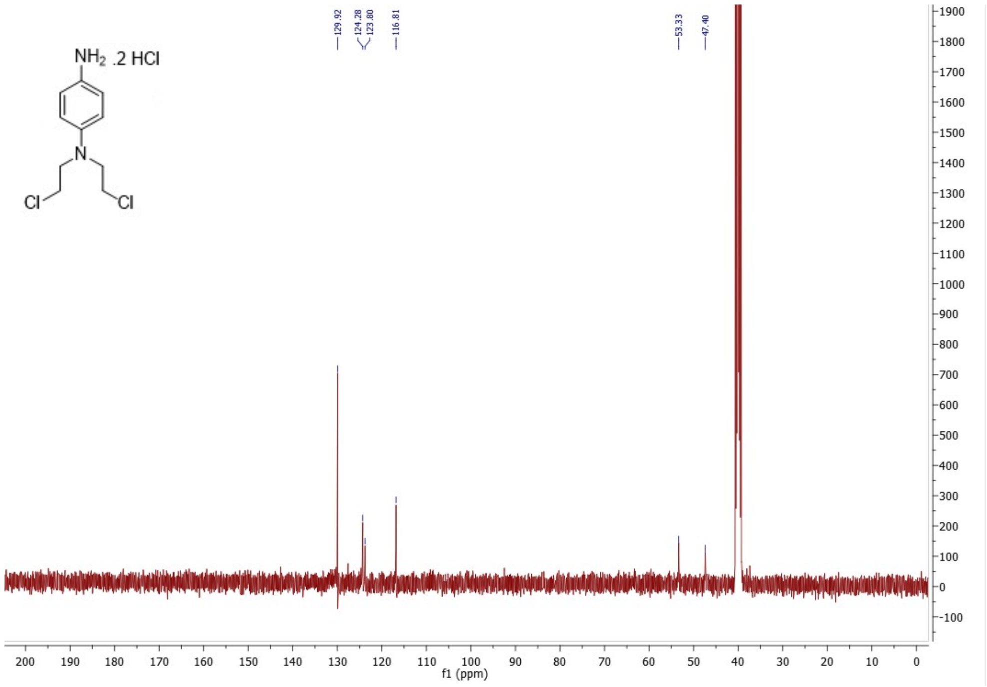

^{13}C NMR spectrum (100 MHz, DMSO-d_6) of compound **9**

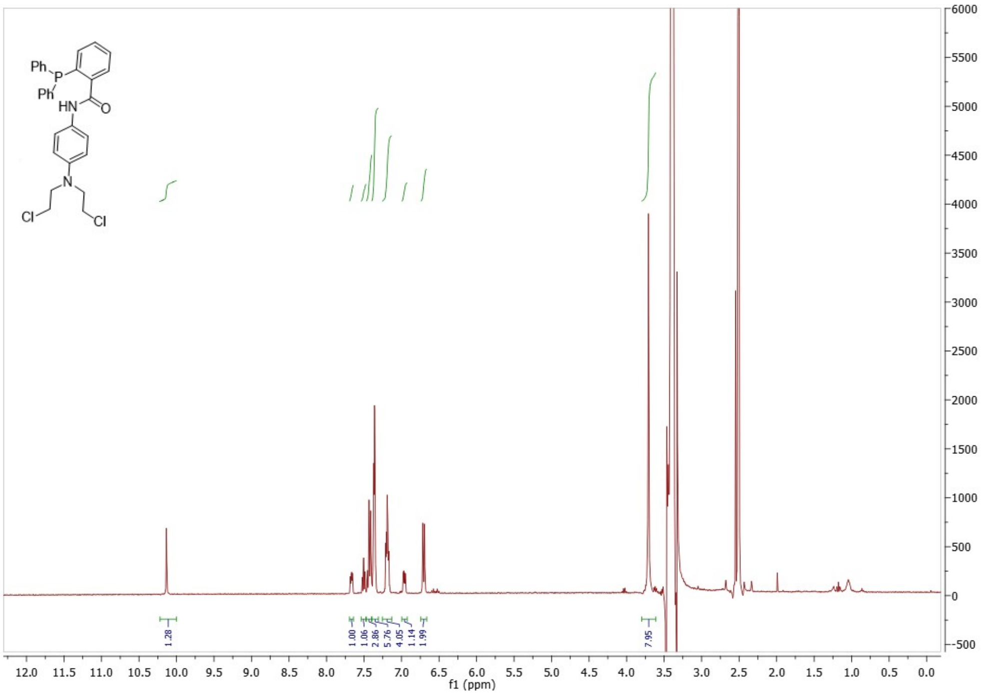

^{1}H NMR spectrum (400 MHz, DMSO-d_6) of compound **10**

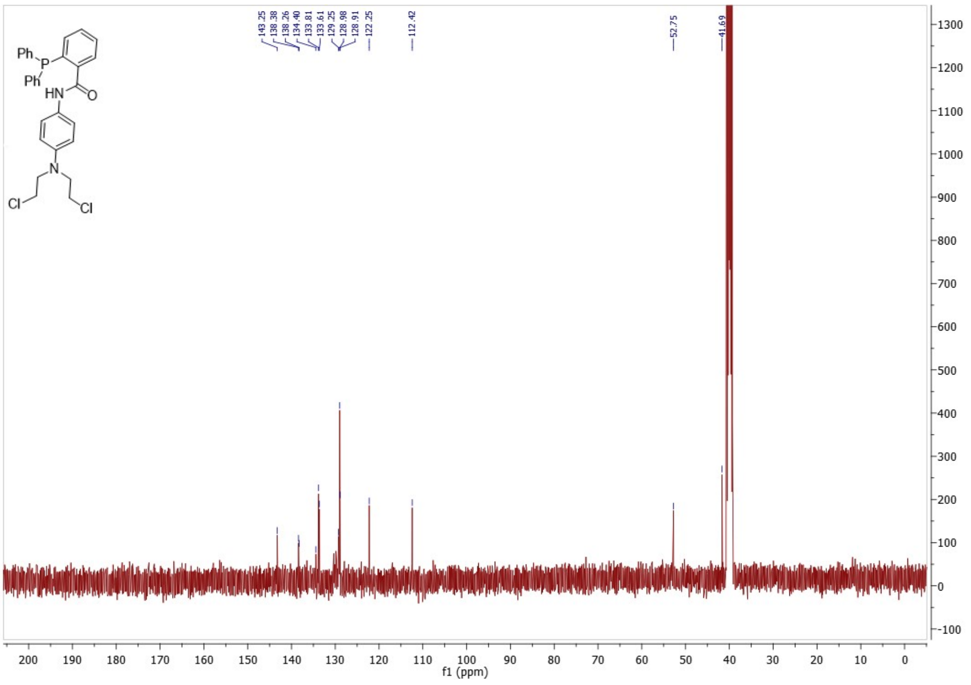

^{13}C NMR spectrum (100 MHz, DMSO-d_6) of compound **10**

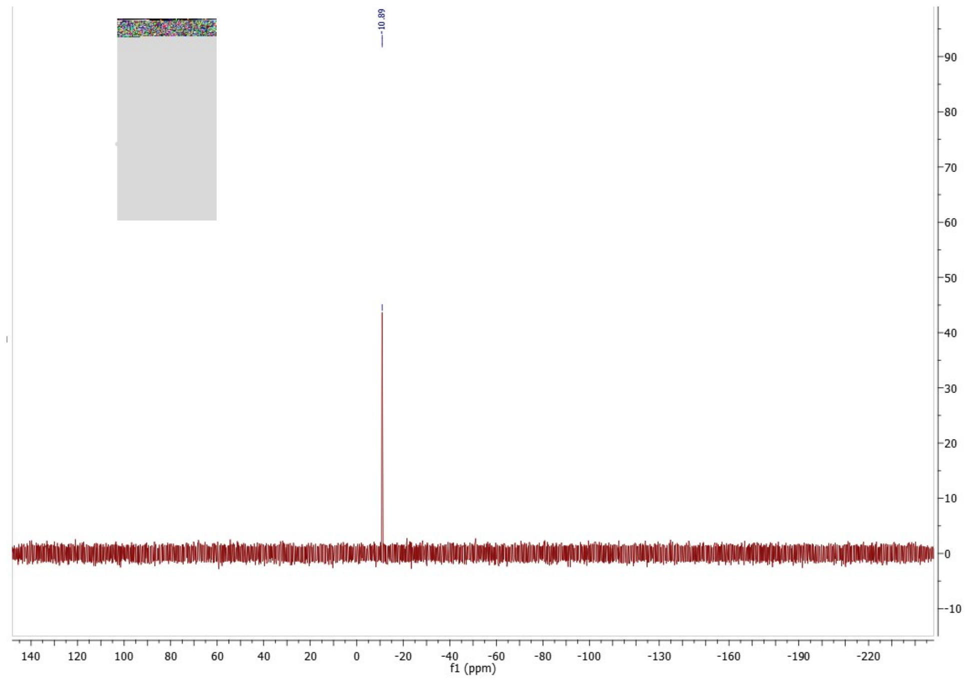

^{31}P NMR spectrum (162 MHz, DMSO-d_6) of compound **10**

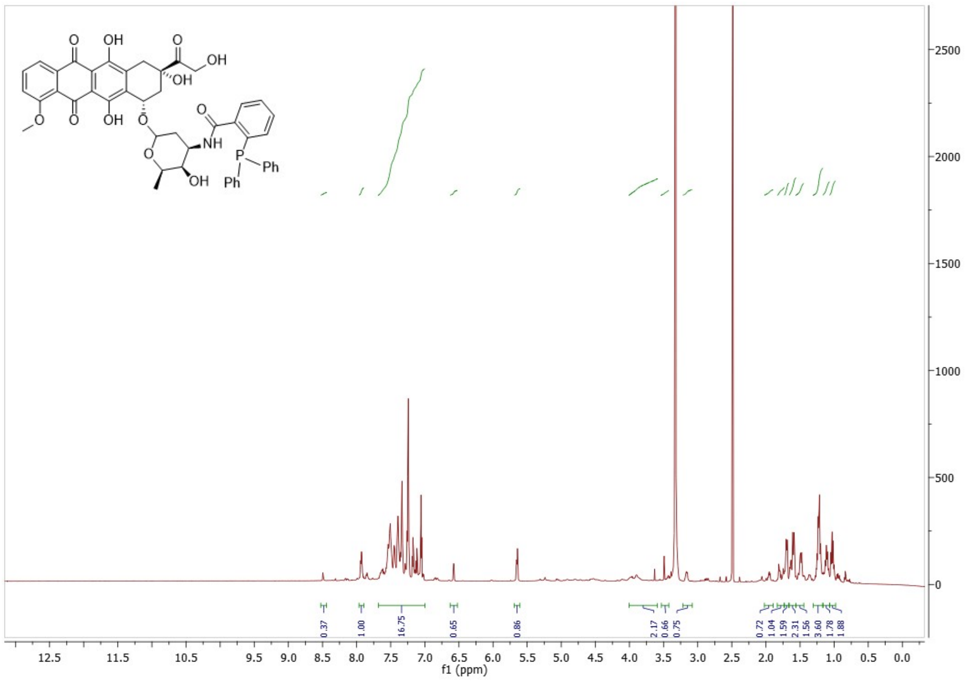

^{1}H NMR spectrum (700 MHz, DMSO-d_6) of compound **12**

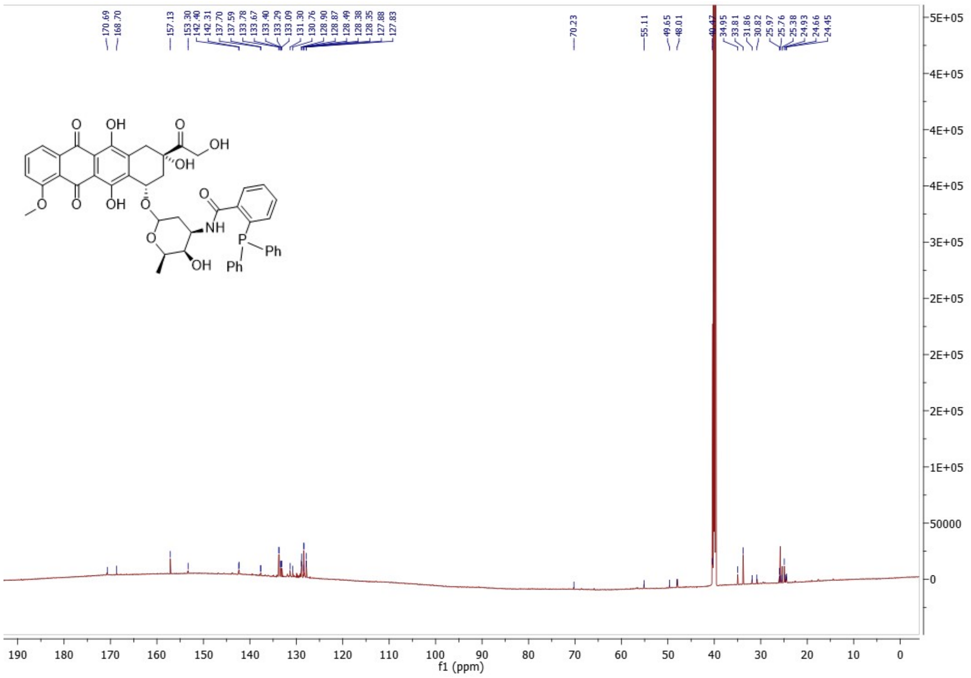

^{13}C NMR spectrum (176 MHz, DMSO-d_6) of compound **12**

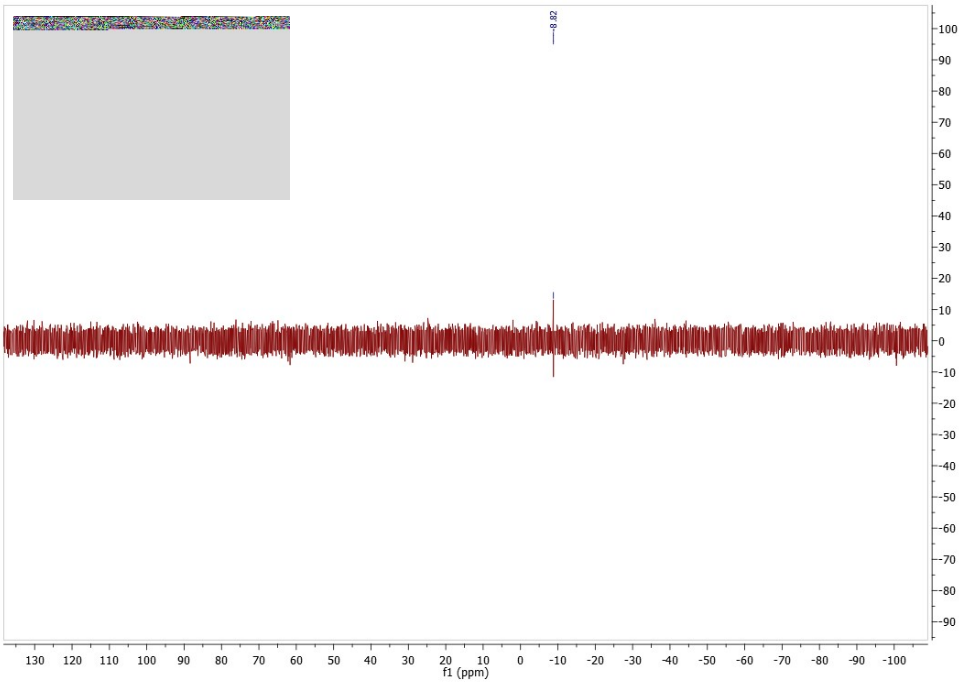

^{31}P NMR spectrum (162 MHz, DMSO-d_6) of compound **12**

HPLC traces of final compounds **4, 5, 10 and 12**

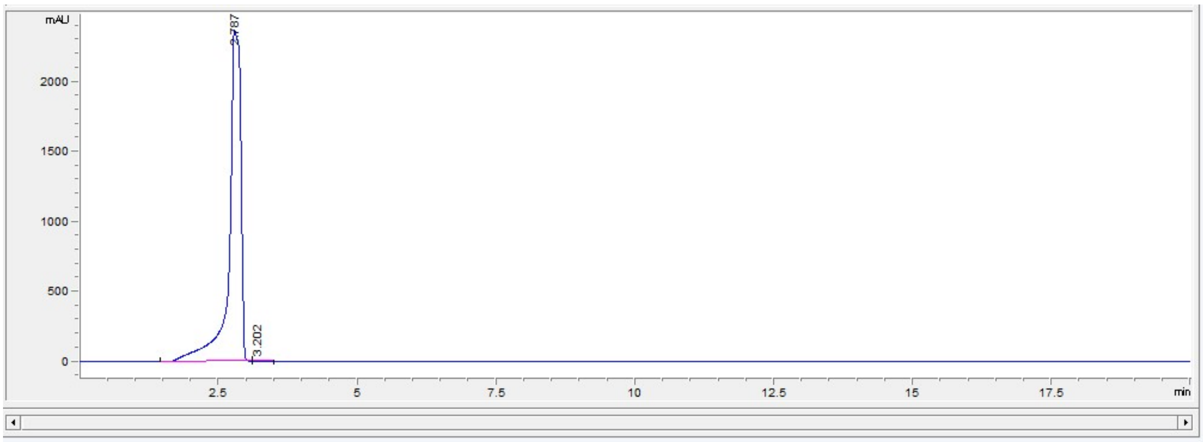

#	Time	Area	Height	Width	Area%	Symmetry
1	2.787	35096.9	2368.6	0.1969	99.897	1.062
2	3.202	36.2	3.2	0.1481	0.103	0.52

The HPLC trace of compound **4**

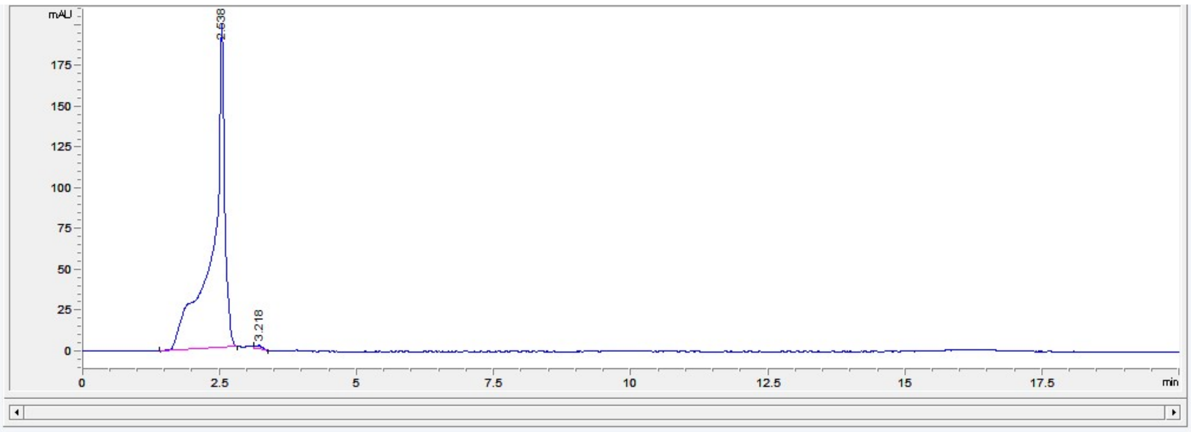

#	Time	Area	Height	Width	Area%	Symmetry
1	2.538	3046.9	198.5	0.197	99.345	3.215
2	3.218	20.1	2.3	0.1138	0.655	0.954

The HPLC trace of compound **5**

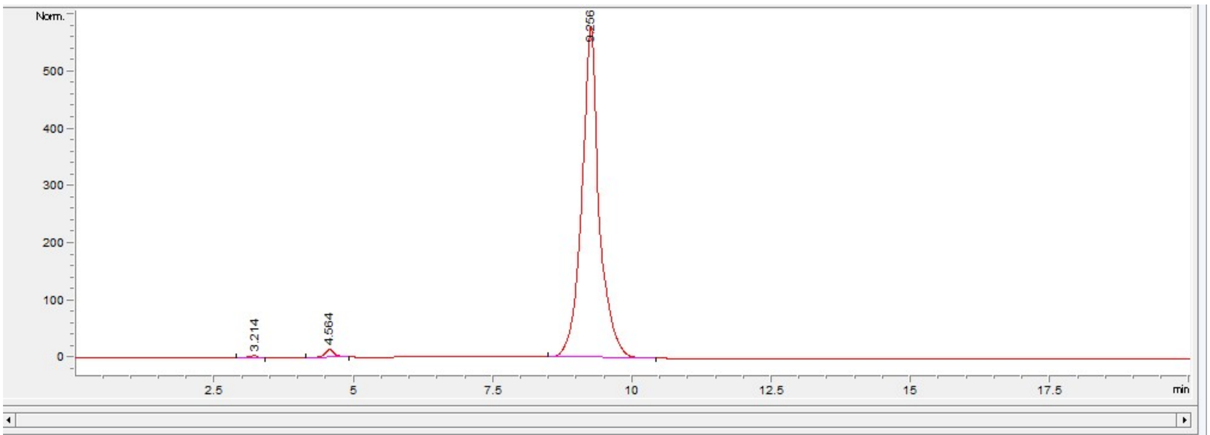

#	Time	Area	Height	Width	Area%	Symmetry
1	3.214	47.8	4.4	0.1479	0.372	1.896
2	4.564	187.7	14.4	0.1819	1.462	0.871
3	9.256	12603.1	578.6	0.3061	98.166	1.026

The HPLC trace of compound **10**

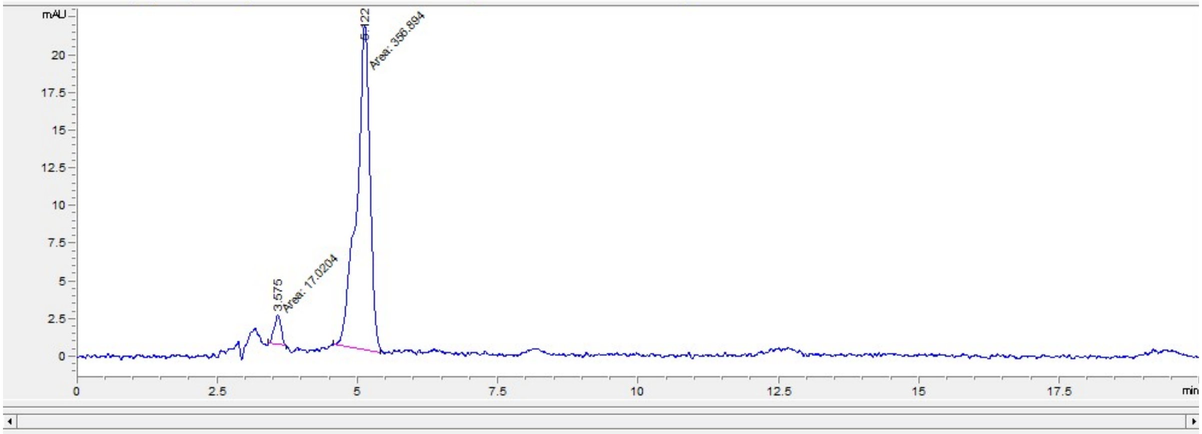

#	Time	Area	Height	Width	Area%	Symmetry
1	3.575	17	2	0.1432	4.552	1.111
2	5.122	356.9	21.6	0.2752	95.448	1.506

The HPLC trace of compound **12**
